# Supplementary figures and images for: Discovery and biological evaluation of a potent small molecule CRM1 inhibitor for its selective ablation of extranodal NK/T cell lymphoma
Source: eLife. 2023 Oct 27;12:e80625. doi: 10.7554/eLife.80625 (PMC10637774; doi:10.7554/eLife.80625)

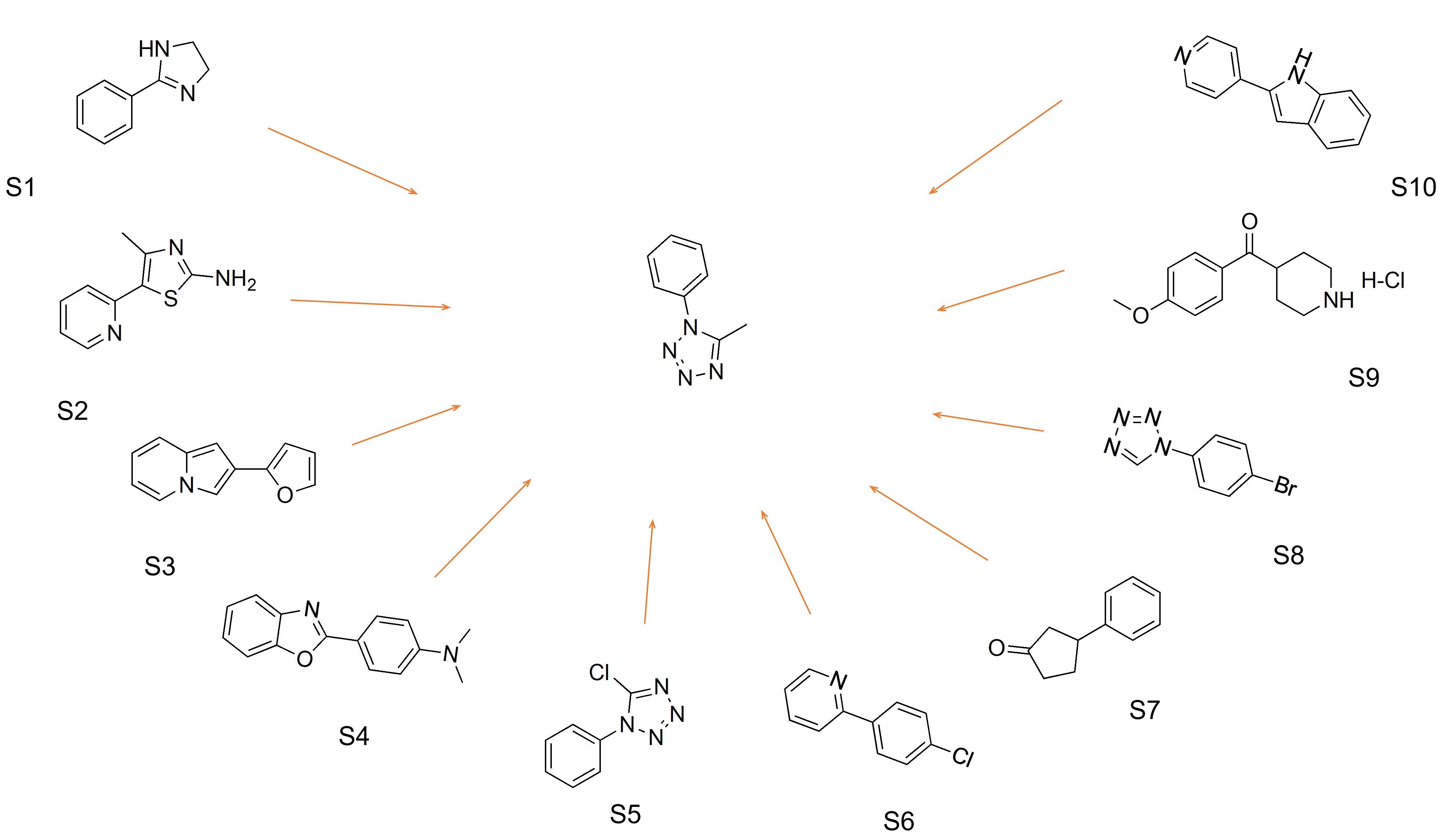

Supplement: Figure 1—source data 1. [file elife-80625-fig1-data1.zip › Figure 1-source data A/Figure1-source data A.jpg]

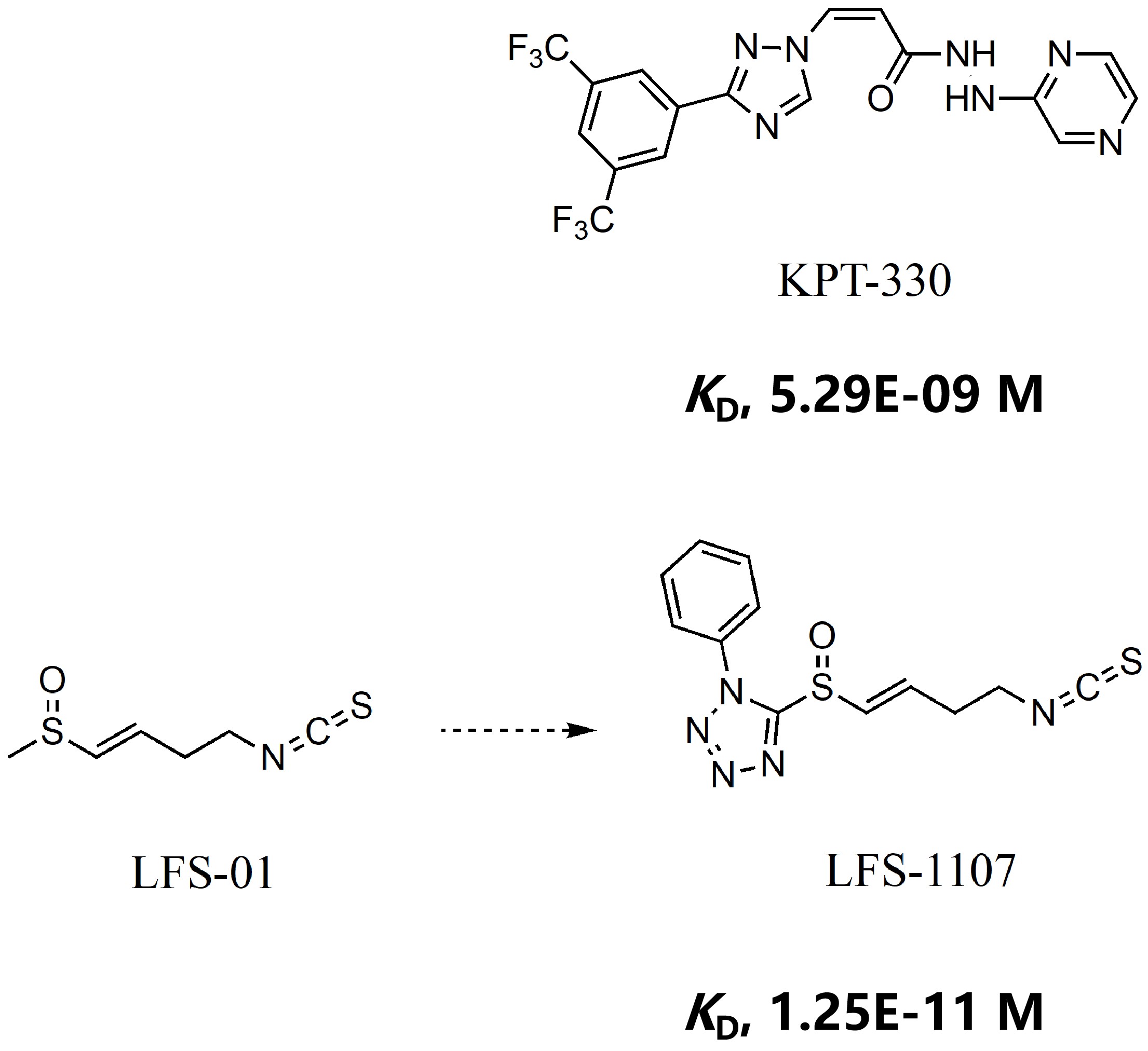

Supplement: Figure 1—source data 2. [file elife-80625-fig1-data2.zip › Figure 1-source data B/Figure1-source data B.jpg]

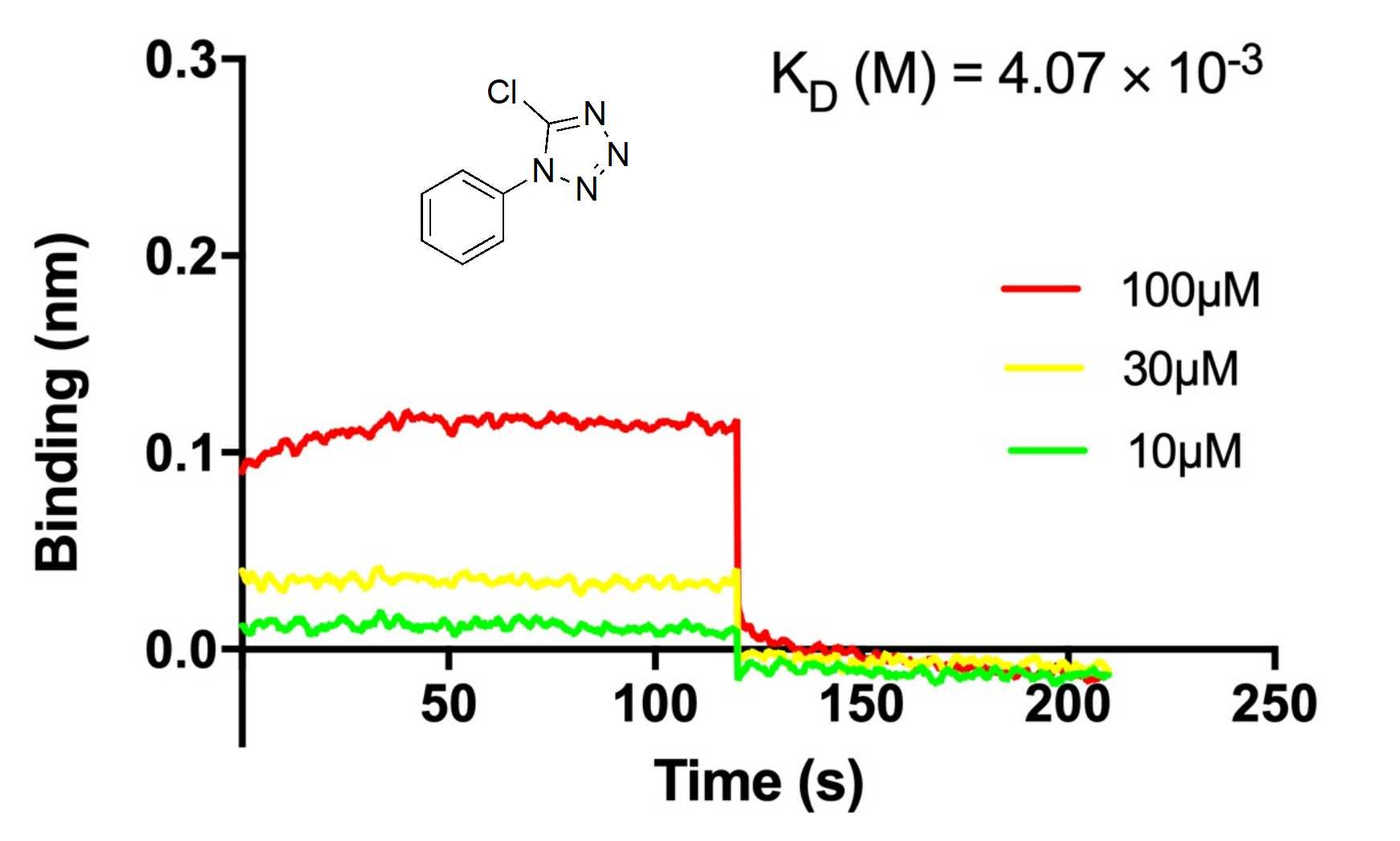

Supplement: Figure 1—source data 3. [file elife-80625-fig1-data3.zip › Figure 1 - source data 3/S5.jpg]

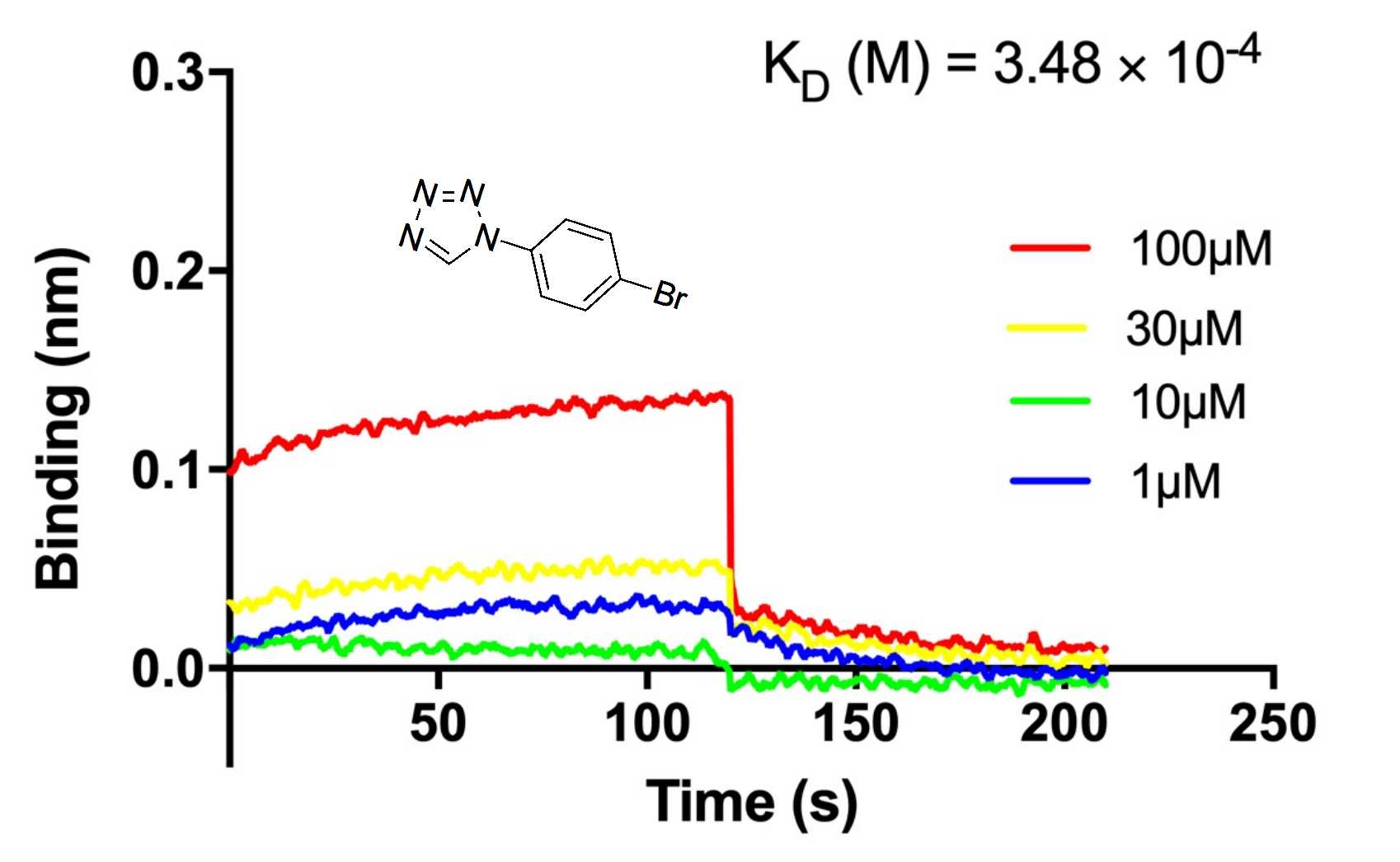

Supplement: Figure 1—source data 3. [file elife-80625-fig1-data3.zip › Figure 1 - source data 3/S8.jpg]

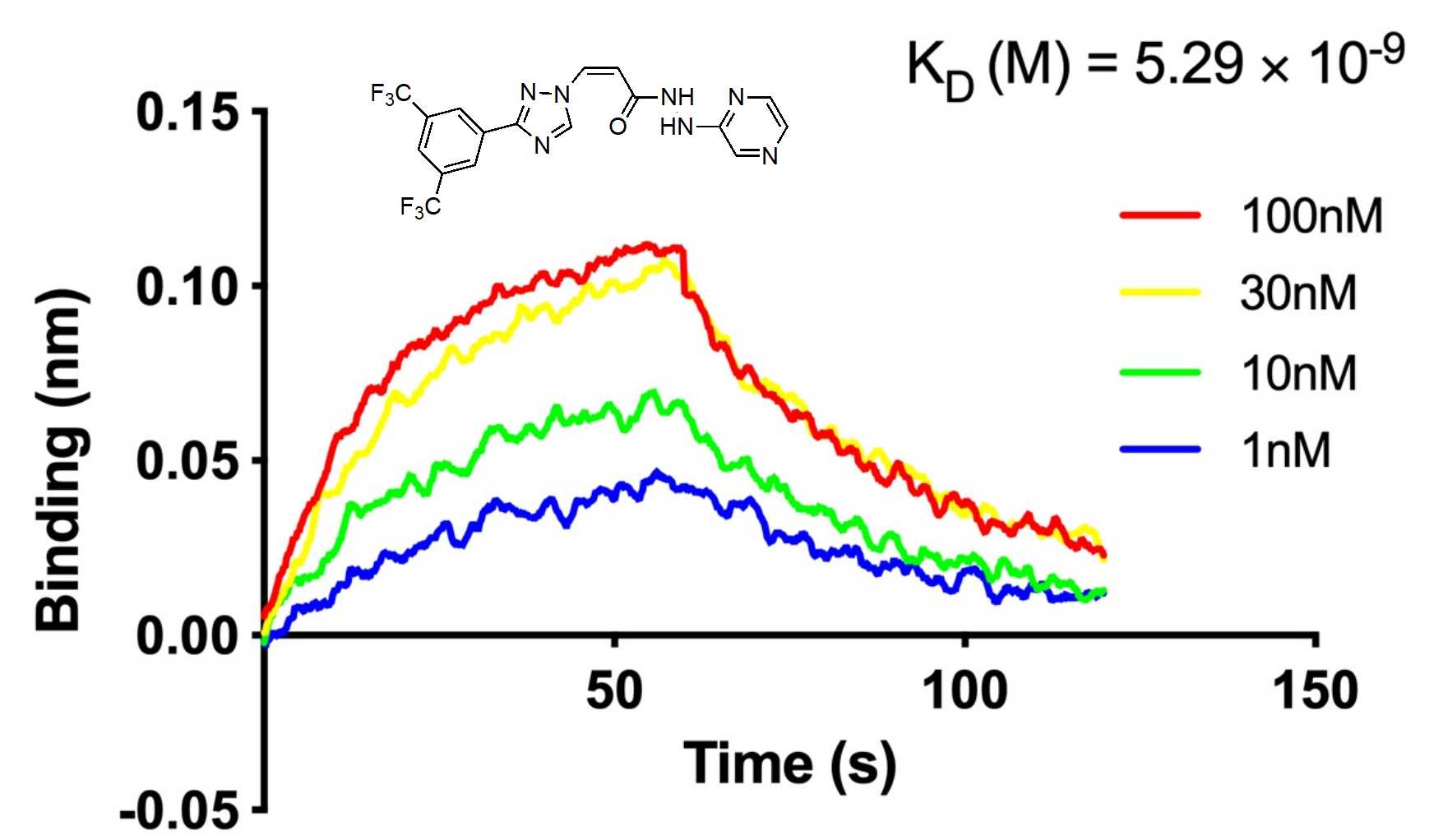

Supplement: Figure 1—source data 4. [file elife-80625-fig1-data4.zip › Figure 1 - source data 4/KPT-330.jpg]

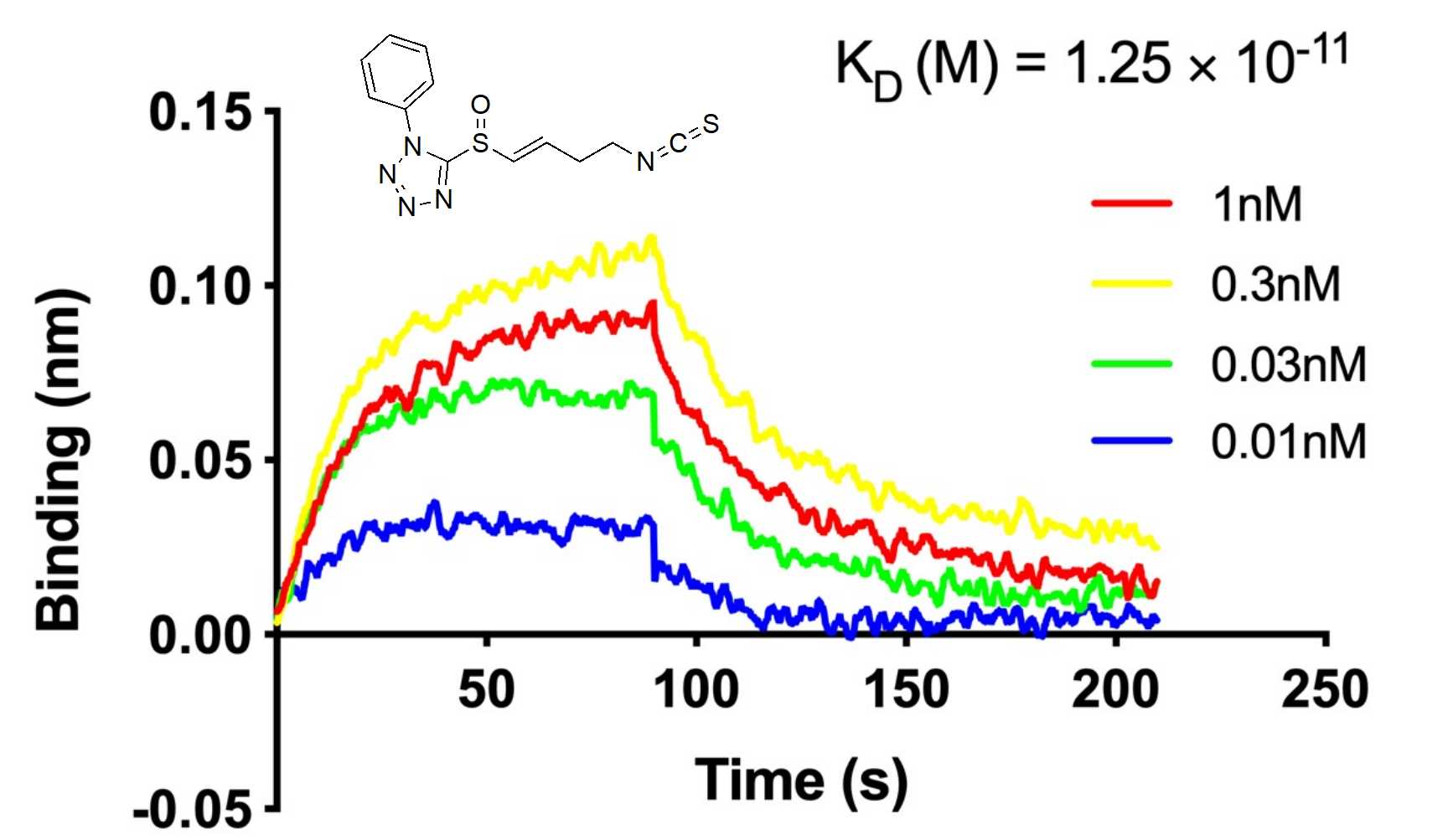

Supplement: Figure 1—source data 4. [file elife-80625-fig1-data4.zip › Figure 1 - source data 4/LFS-1107.jpg]

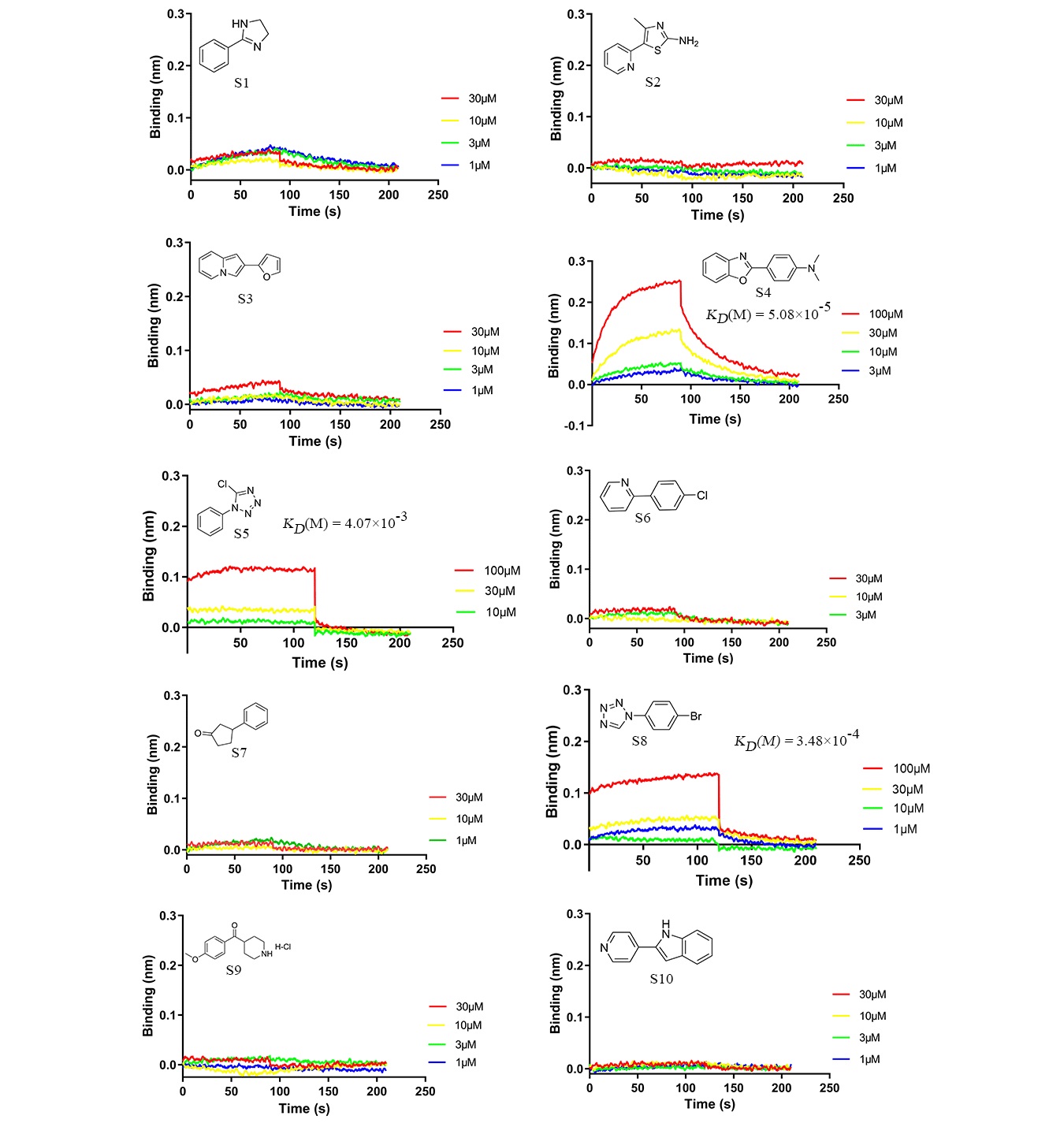

Supplement: Figure 1—figure supplement 1—source data 1. [file elife-80625-fig1-figsupp1-data1.zip › Figure 1 - figure supplement 1 - source data/Figure 1 - Figure suppement 1.jpg]

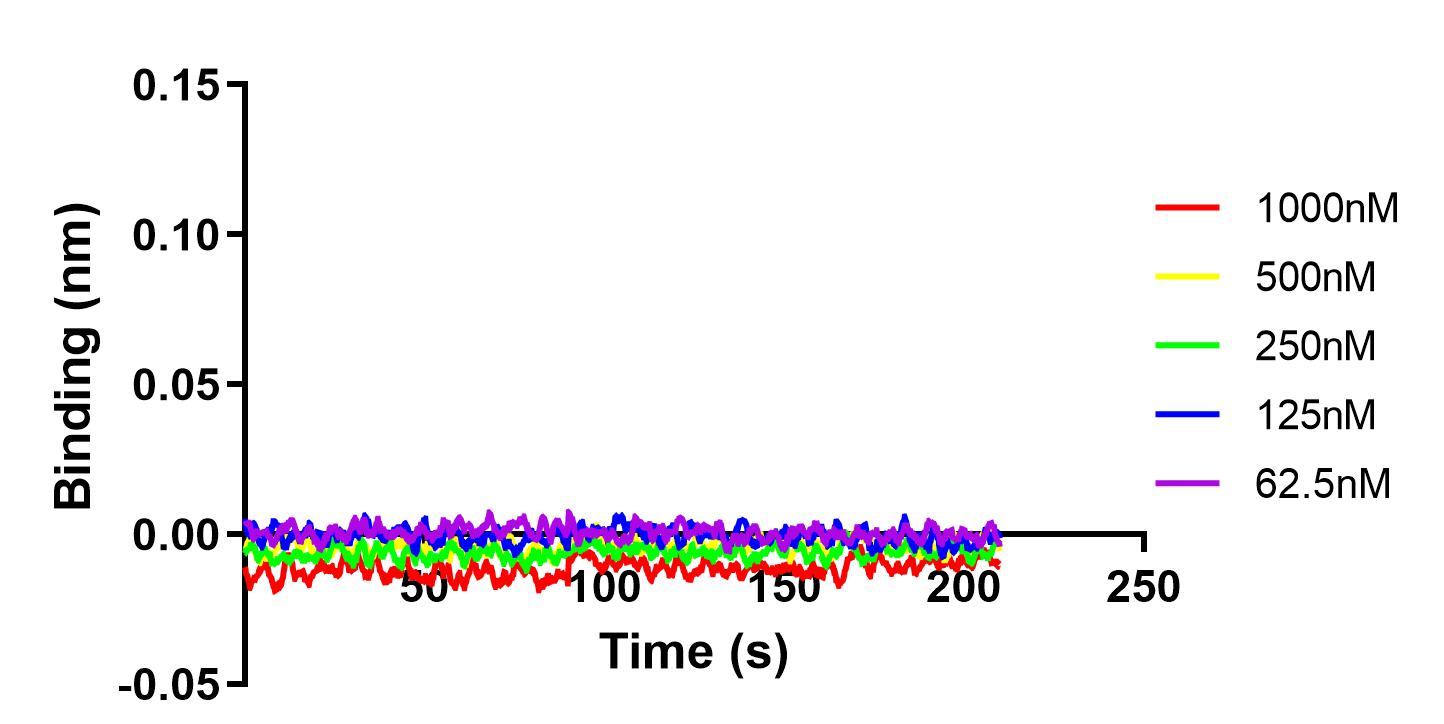

Supplement: Figure 1—figure supplement 2—source data 1. [file elife-80625-fig1-figsupp2-data1.zip › Figure 1 - figure supplement 2 - source data/B.jpg]

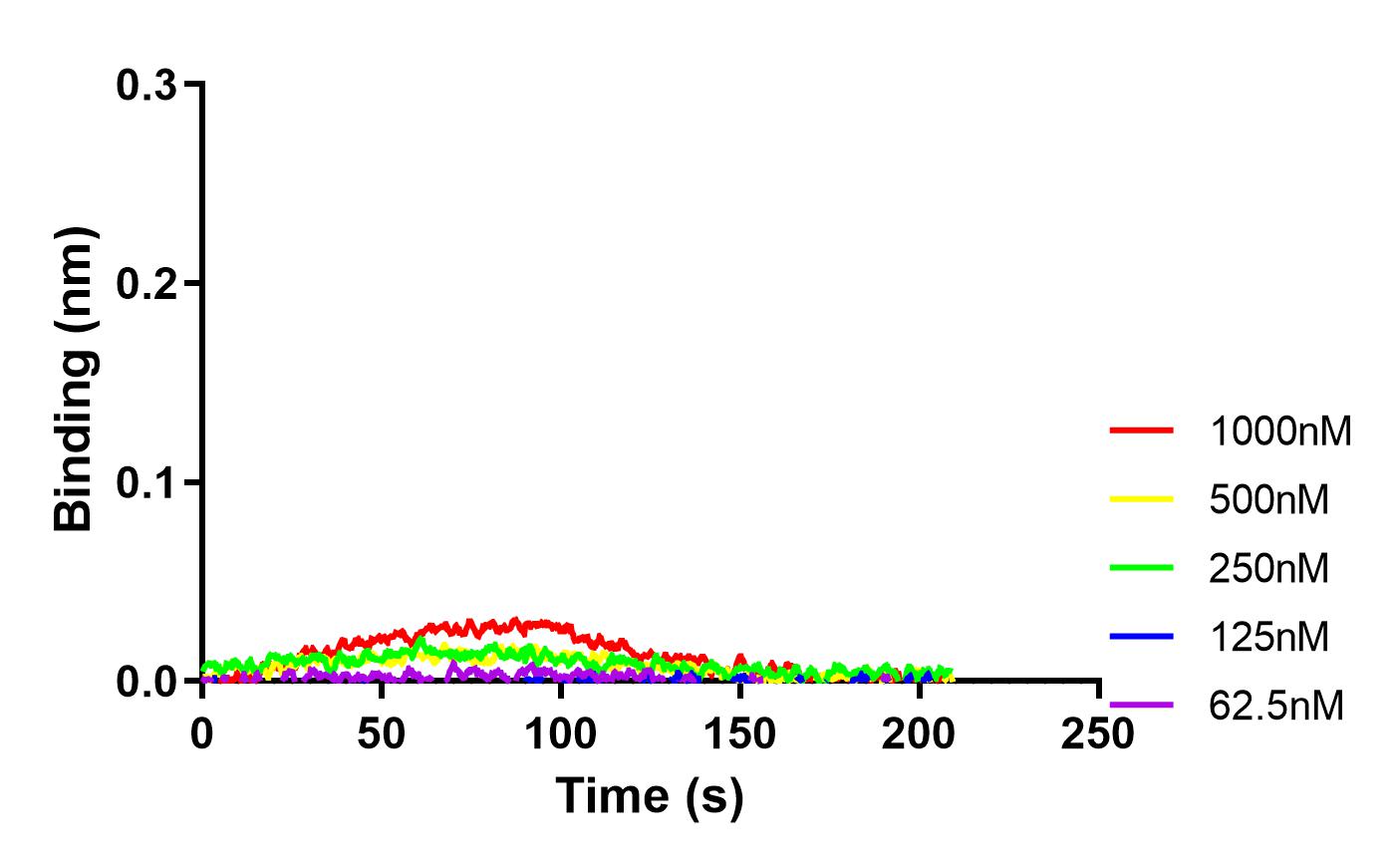

Supplement: Figure 1—figure supplement 2—source data 1. [file elife-80625-fig1-figsupp2-data1.zip › Figure 1 - figure supplement 2 - source data/A.jpg]

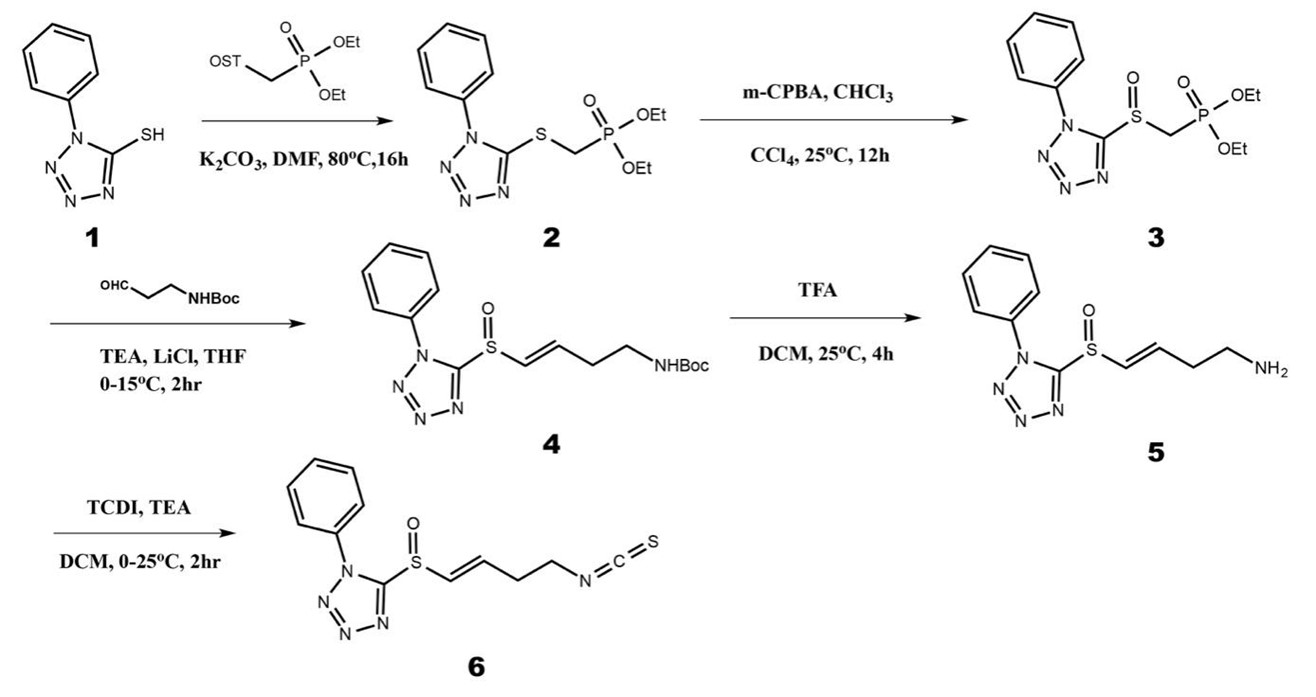

Supplement: Figure 1—figure supplement 3—source data 1. [file elife-80625-fig1-figsupp3-data1.zip › Figure 1-figure supplement 3-source data/Scheme.jpg]

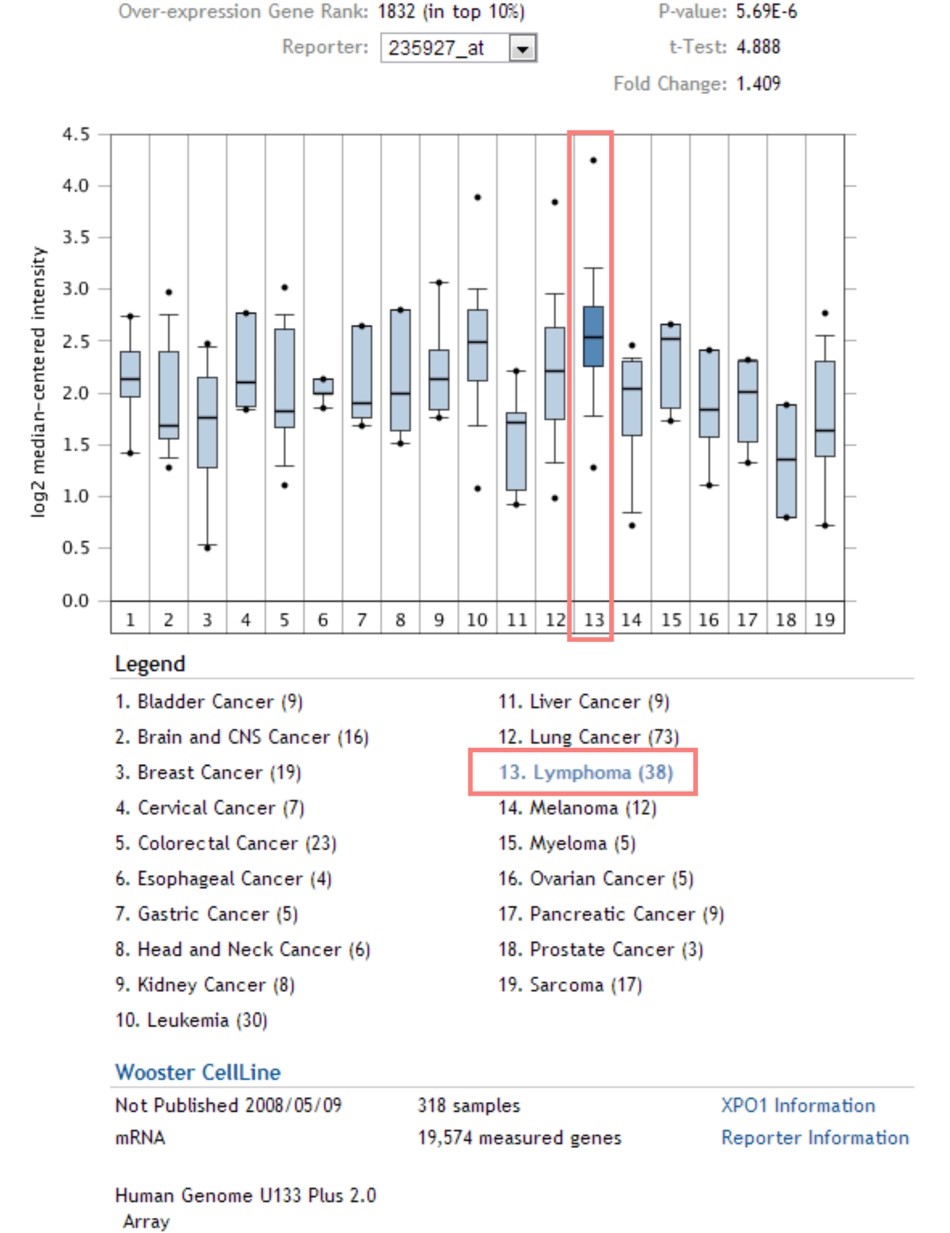

Supplement: Figure 1—figure supplement 4—source data 1. [file elife-80625-fig1-figsupp4-data1.zip › Figure 1-figure supplement 4-source data/S4.jpg]

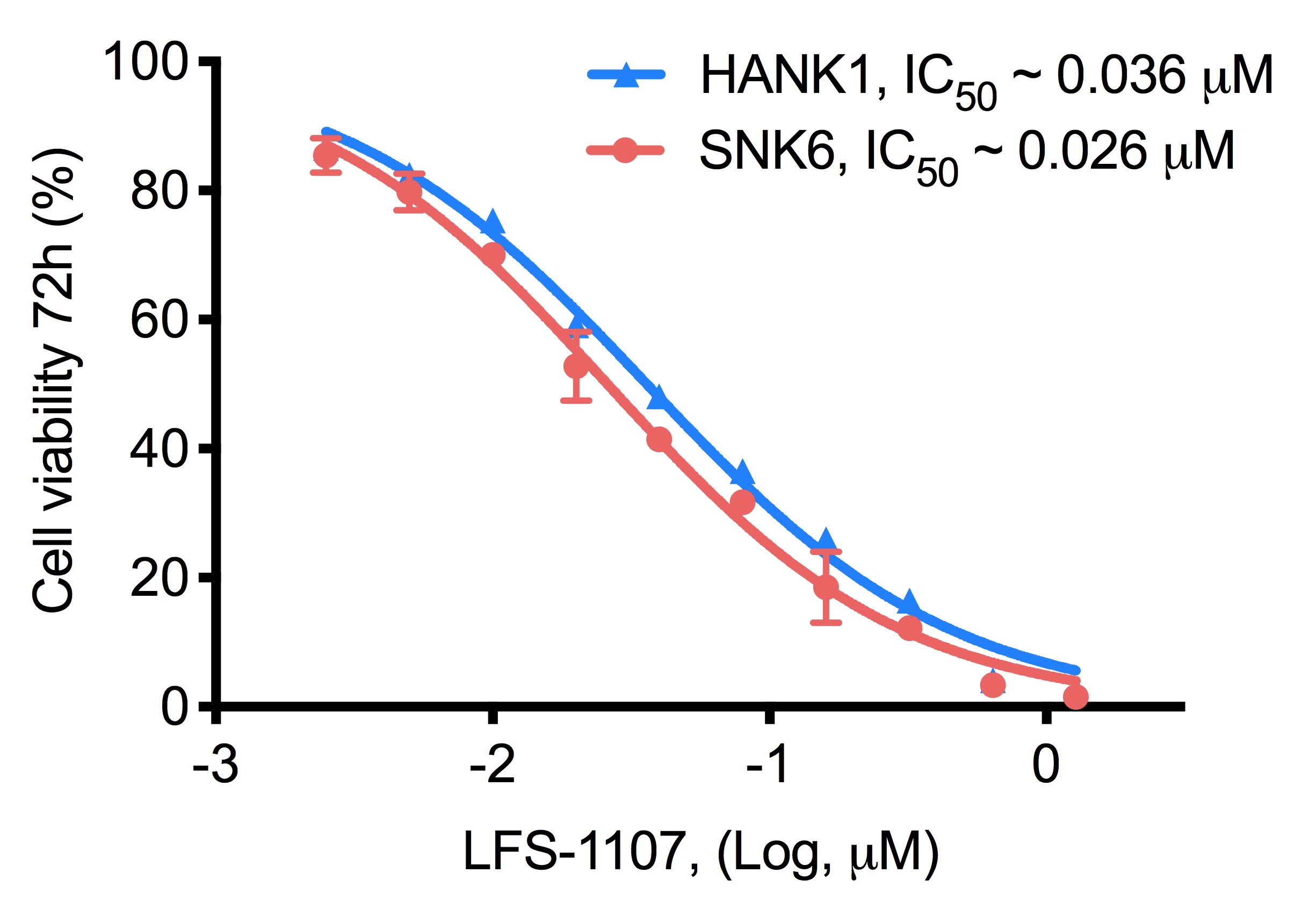

Supplement: Figure 2—source data 1. [file elife-80625-fig2-data1.zip › Figure 2 - source data 1/A.jpg]

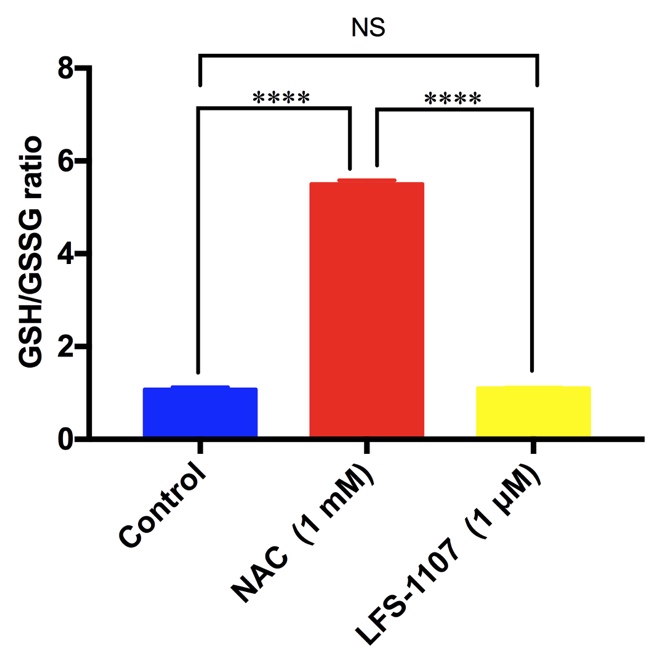

Supplement: Figure 2—source data 2. [file elife-80625-fig2-data2.zip › Figure 2 - source data 2/B.jpg]

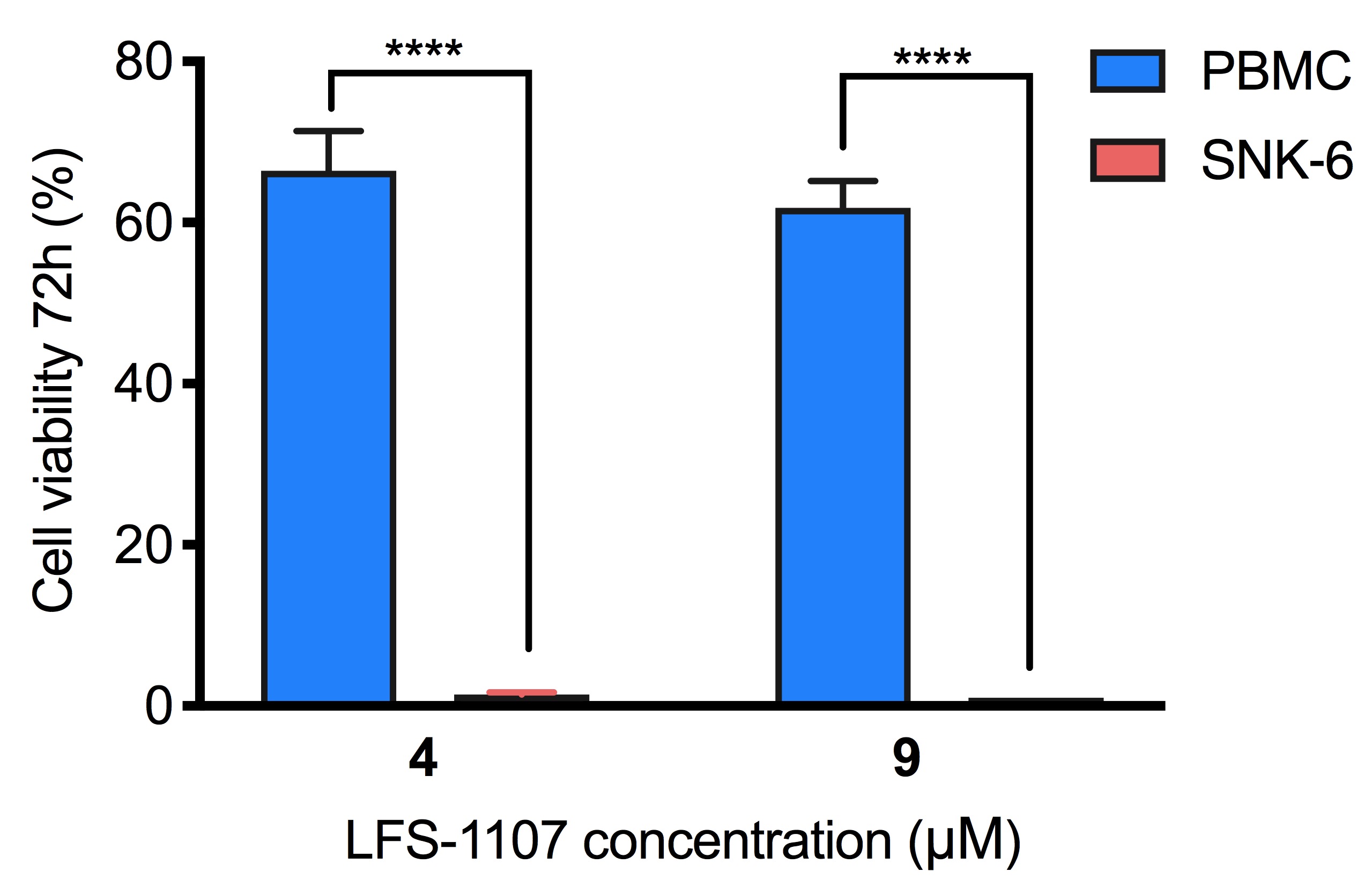

Supplement: Figure 2—source data 3. [file elife-80625-fig2-data3.zip › Figure 2 - source data 3/C.jpg]

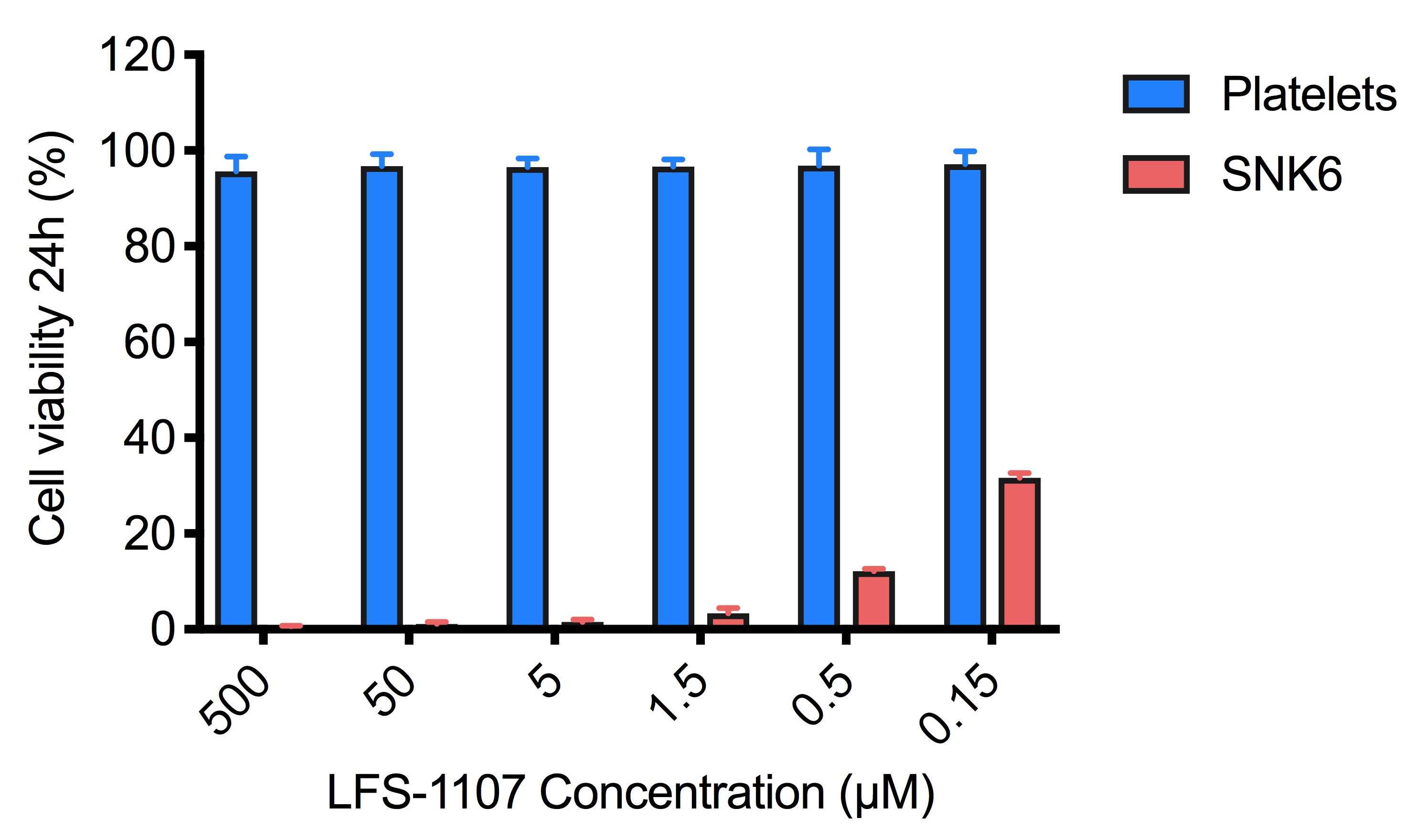

Supplement: Figure 2—source data 4. [file elife-80625-fig2-data4.zip › Figure 2-source data 4/D.jpg]

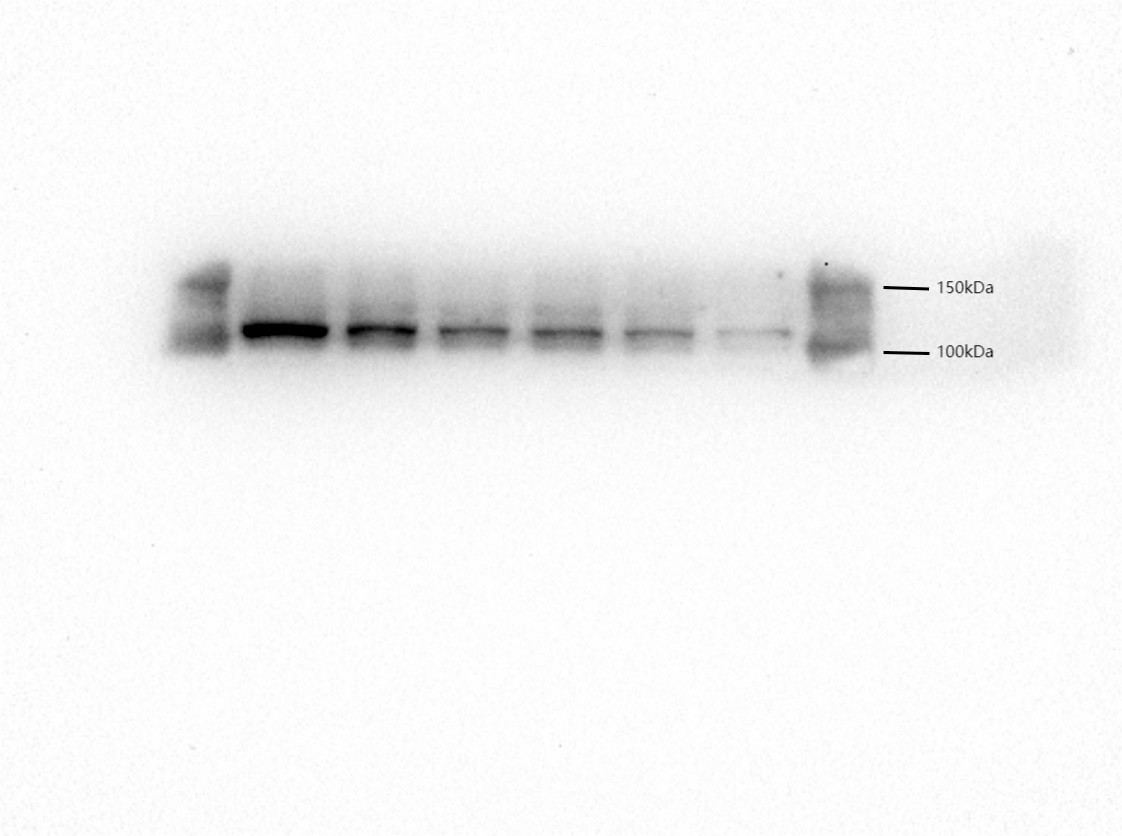

Supplement: Figure 2—source data 5. [file elife-80625-fig2-data5.zip › Figure 2-source data 5/CRM1_A.jpg]

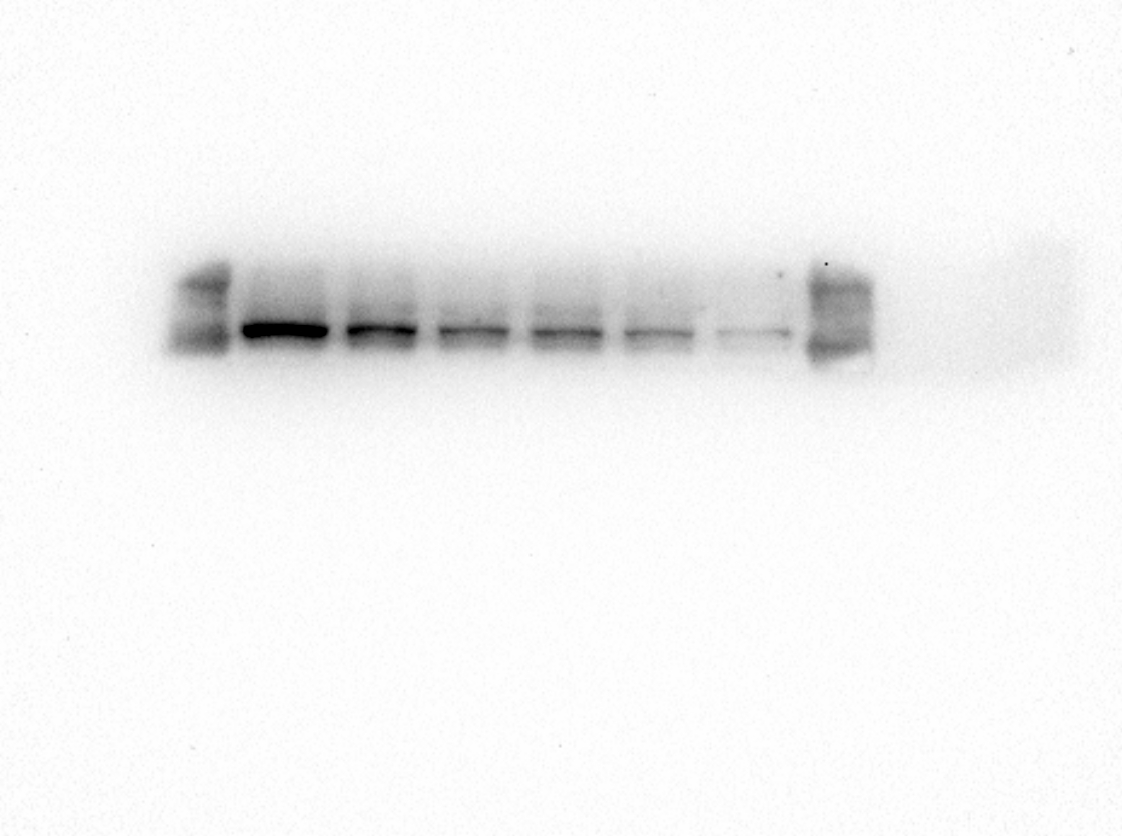

Supplement: Figure 2—source data 5. [file elife-80625-fig2-data5.zip › Figure 2-source data 5/CRM1_B.jpg]

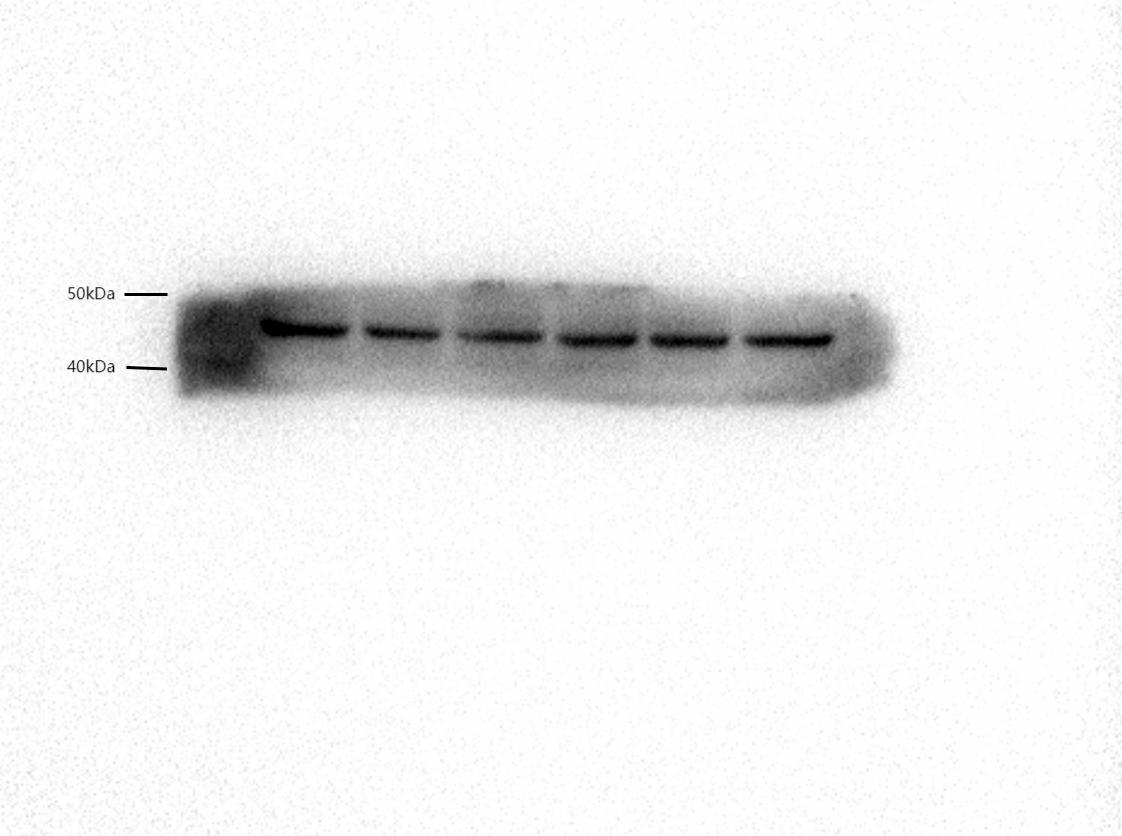

Supplement: Figure 2—source data 5. [file elife-80625-fig2-data5.zip › Figure 2-source data 5/a┬-actin_A.jpg]

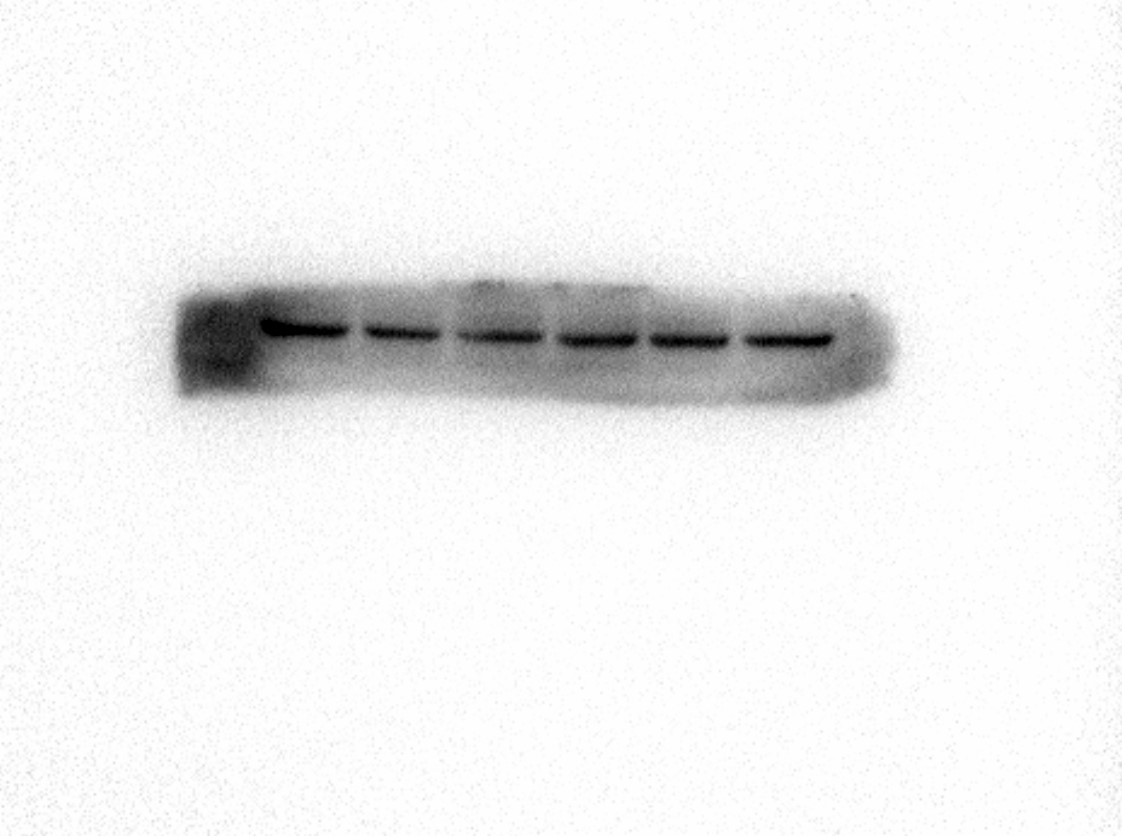

Supplement: Figure 2—source data 5. [file elife-80625-fig2-data5.zip › Figure 2-source data 5/a┬-actin_B.jpg]

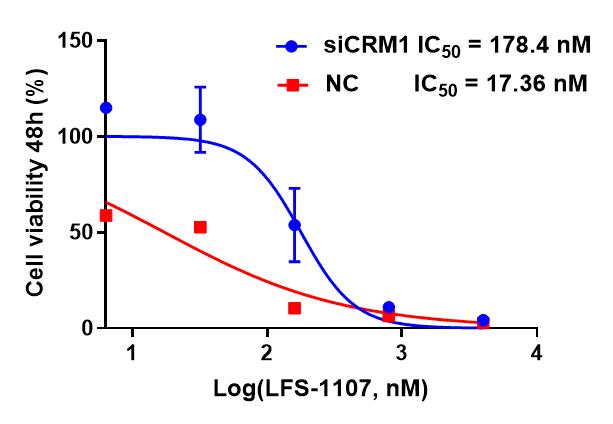

Supplement: Figure 2—source data 6. [file elife-80625-fig2-data6.zip › Figure 2-source data 6/F.jpg]

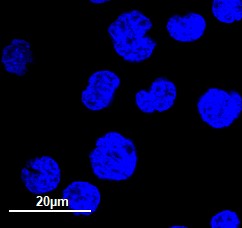

Supplement: Figure 2—source data 7. [file elife-80625-fig2-data7.zip › Figure 2-source data G/DAPI/DMSO.jpg]

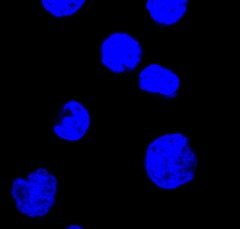

Supplement: Figure 2—source data 7. [file elife-80625-fig2-data7.zip › Figure 2-source data G/DAPI/KPT-330(200nM).jpg]

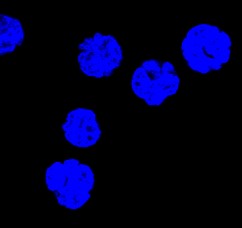

Supplement: Figure 2—source data 7. [file elife-80625-fig2-data7.zip › Figure 2-source data G/DAPI/LFS-1107(100nM).jpg]

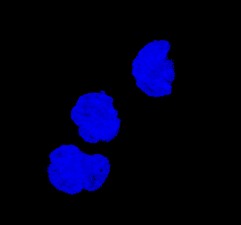

Supplement: Figure 2—source data 7. [file elife-80625-fig2-data7.zip › Figure 2-source data G/DAPI/LFS-1107(200nM).jpg]

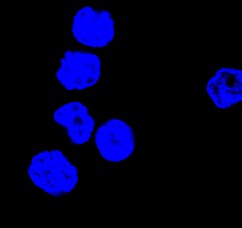

Supplement: Figure 2—source data 7. [file elife-80625-fig2-data7.zip › Figure 2-source data G/DAPI/LFS-1107(50nM).jpg]

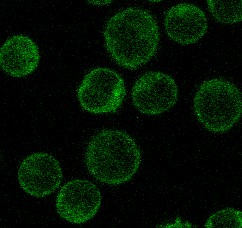

Supplement: Figure 2—source data 7. [file elife-80625-fig2-data7.zip › Figure 2-source data G/Ia╩Ba┴/DMSO.jpg]

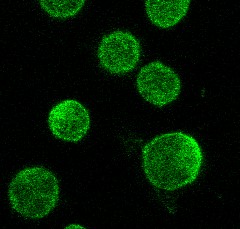

Supplement: Figure 2—source data 7. [file elife-80625-fig2-data7.zip › Figure 2-source data G/Ia╩Ba┴/KPT-330(200nM).jpg]

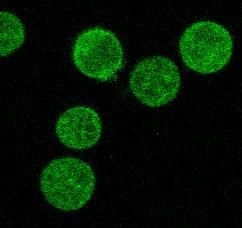

Supplement: Figure 2—source data 7. [file elife-80625-fig2-data7.zip › Figure 2-source data G/Ia╩Ba┴/LFS-1107(100nM).jpg]

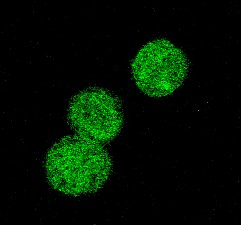

Supplement: Figure 2—source data 7. [file elife-80625-fig2-data7.zip › Figure 2-source data G/Ia╩Ba┴/LFS-1107(200nM).jpg]

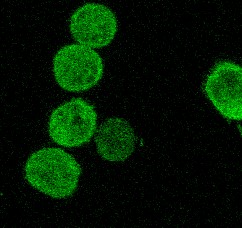

Supplement: Figure 2—source data 7. [file elife-80625-fig2-data7.zip › Figure 2-source data G/Ia╩Ba┴/LFS-1107(50nM).jpg]

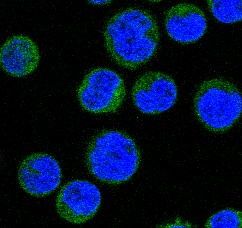

Supplement: Figure 2—source data 7. [file elife-80625-fig2-data7.zip › Figure 2-source data G/Merged/DMSO.jpg]

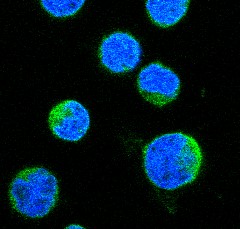

Supplement: Figure 2—source data 7. [file elife-80625-fig2-data7.zip › Figure 2-source data G/Merged/KPT-330(200nM).jpg]

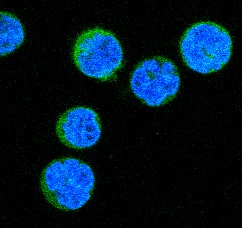

Supplement: Figure 2—source data 7. [file elife-80625-fig2-data7.zip › Figure 2-source data G/Merged/LFS-1107(100nM).jpg]

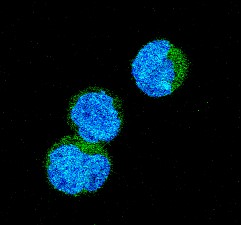

Supplement: Figure 2—source data 7. [file elife-80625-fig2-data7.zip › Figure 2-source data G/Merged/LFS-1107(200nM).jpg]

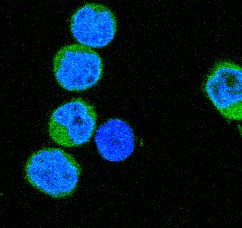

Supplement: Figure 2—source data 7. [file elife-80625-fig2-data7.zip › Figure 2-source data G/Merged/LFS-1107(50nM).jpg]

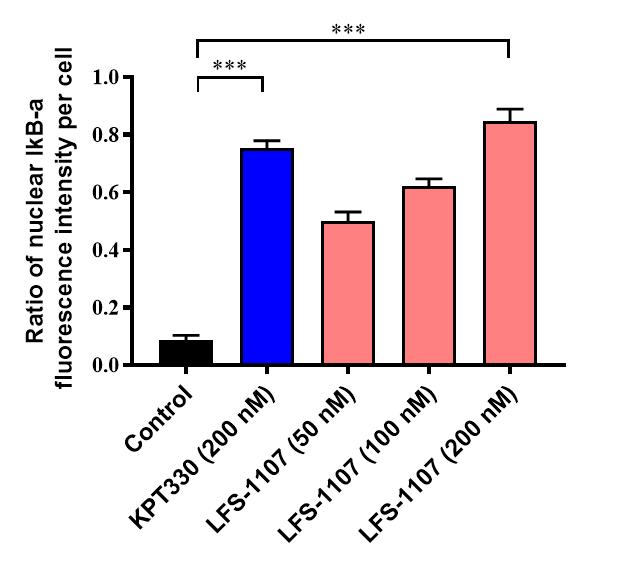

Supplement: Figure 2—source data 8. [file elife-80625-fig2-data8.zip › Figure 2-source data 8/H.jpg]

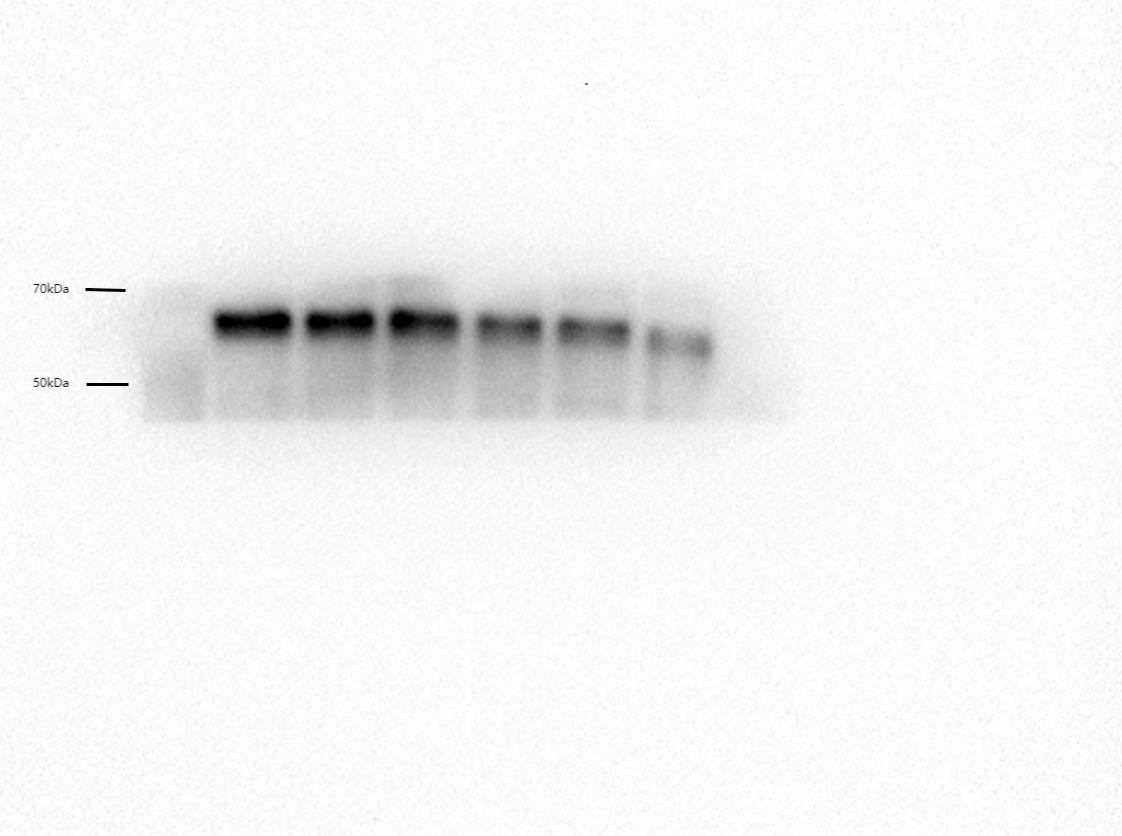

Supplement: Figure 2—source data 9. [file elife-80625-fig2-data9.zip › Figure 2-source data 9/Figure 2-source data I/c-Myc/C-Myc_B.jpg]

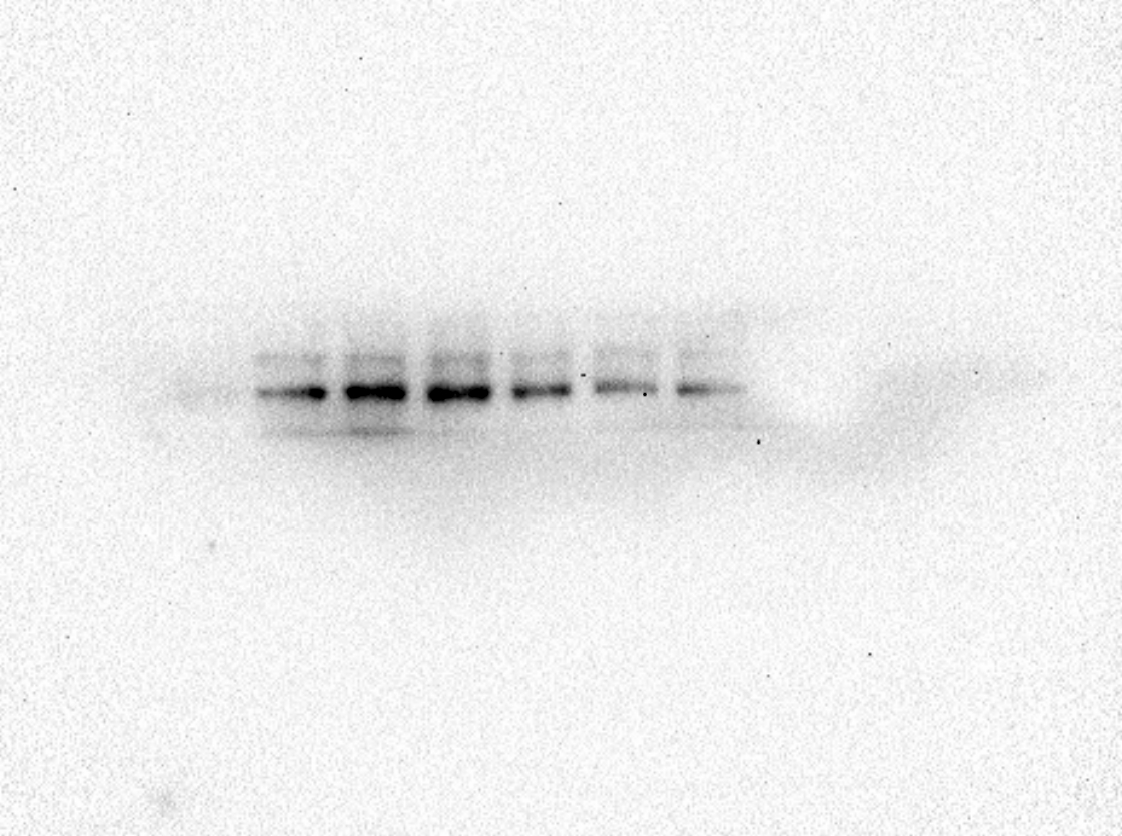

Supplement: Figure 2—source data 9. [file elife-80625-fig2-data9.zip › Figure 2-source data 9/Figure 2-source data I/COX-2/COX-2_B.jpg]

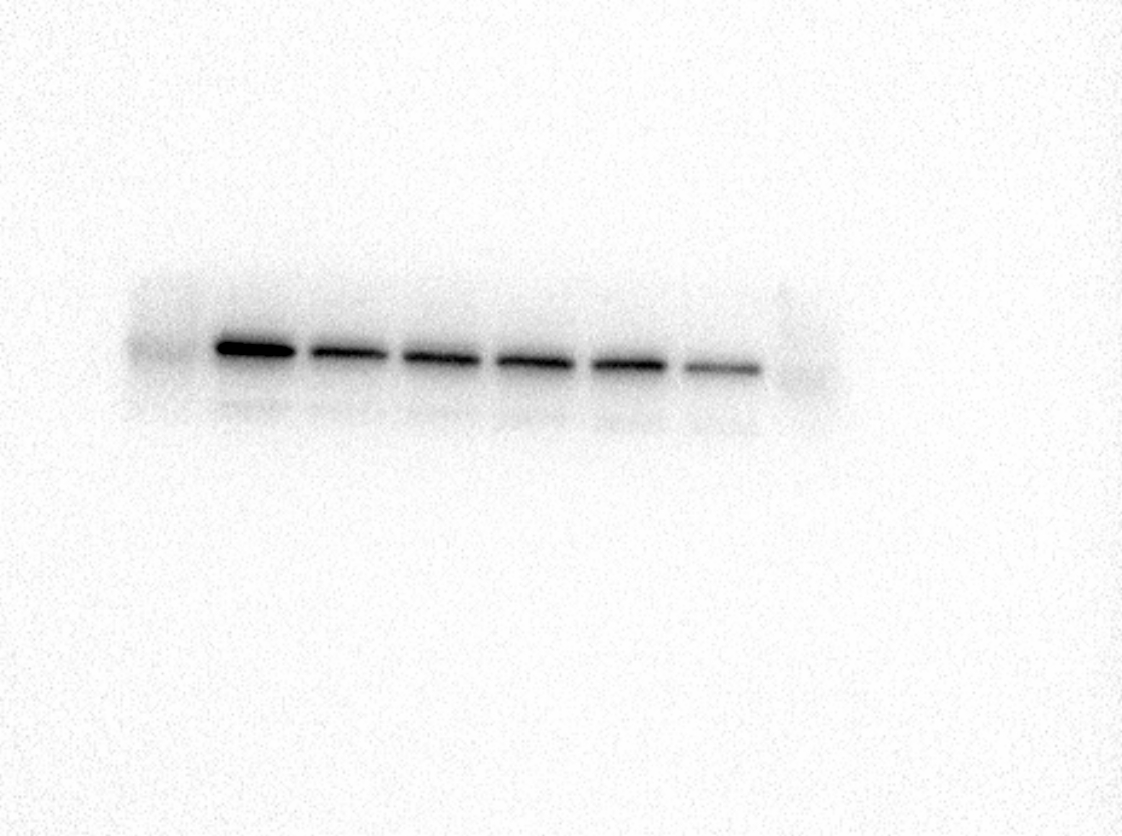

Supplement: Figure 2—source data 9. [file elife-80625-fig2-data9.zip › Figure 2-source data 9/Figure 2-source data I/p65/p65_B.jpg]

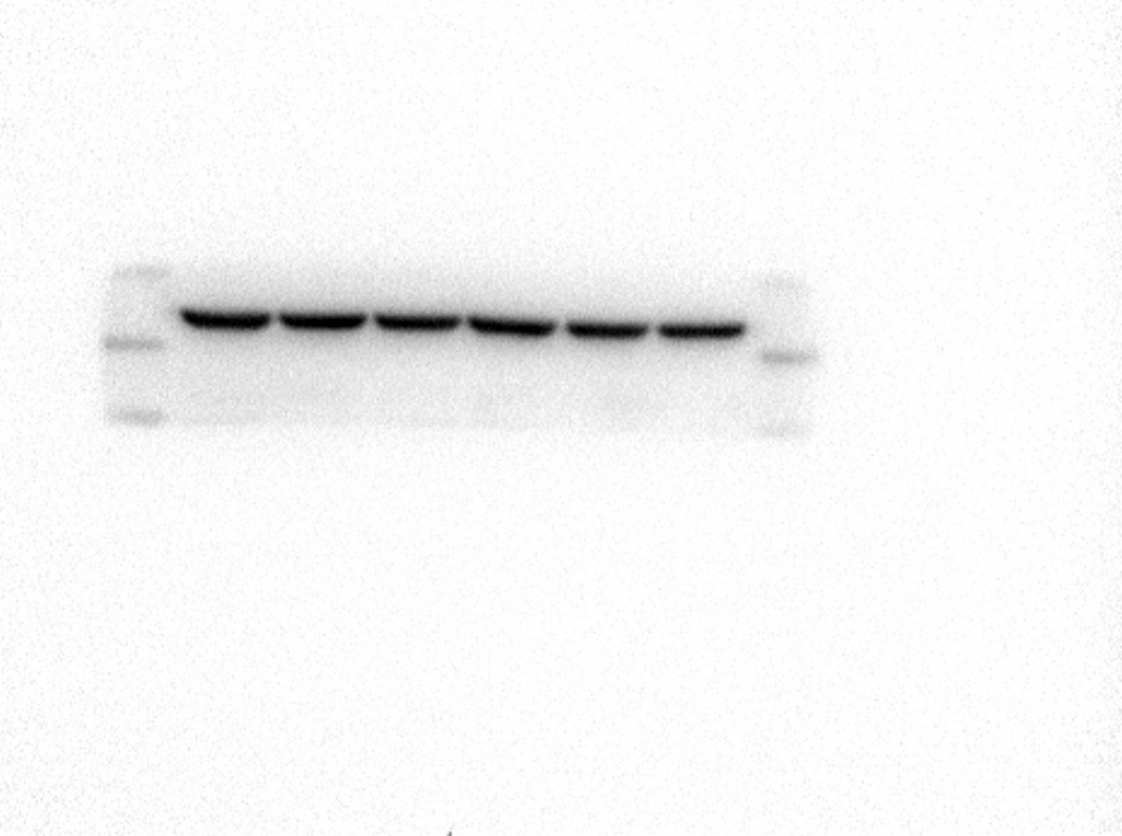

Supplement: Figure 2—source data 9. [file elife-80625-fig2-data9.zip › Figure 2-source data 9/Figure 2-source data I/a┬-actin/a┬-actin_B.jpg]

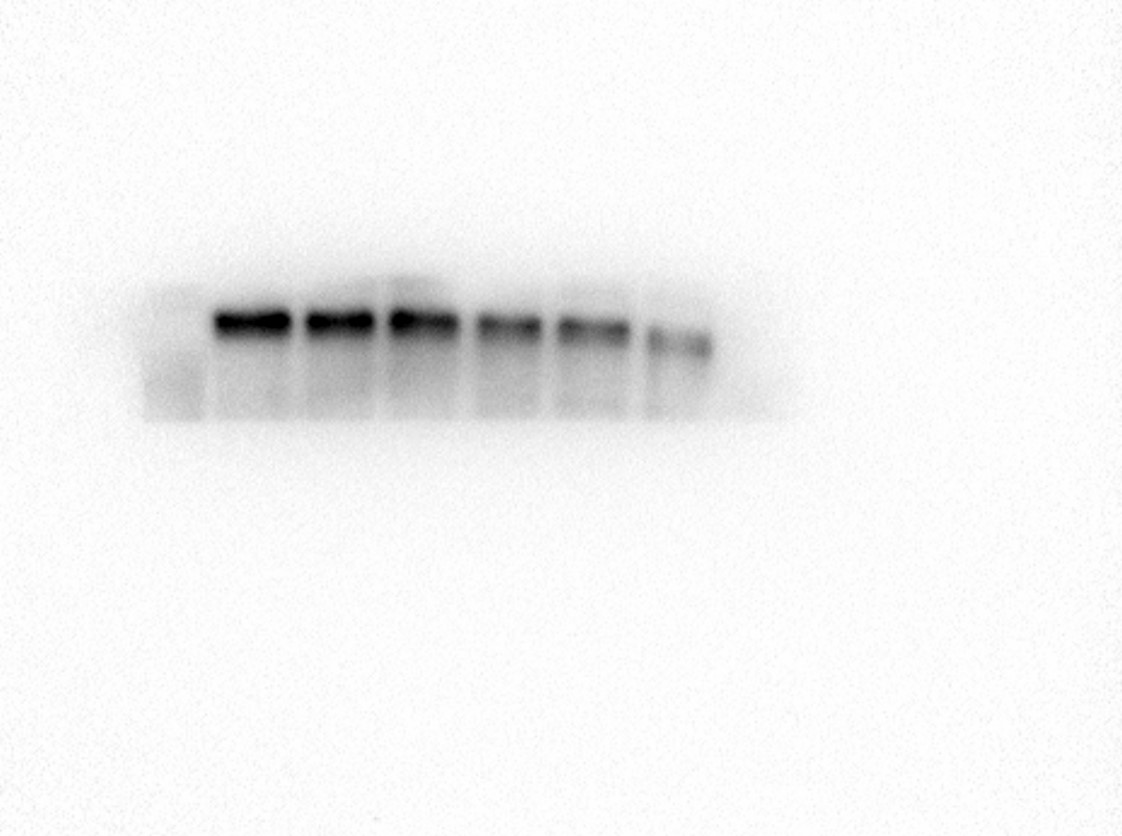

Supplement: Figure 2—source data 9. [file elife-80625-fig2-data9.zip › Figure 2-source data 9/Figure 2-source data I/c-Myc/c-myc_A.jpg]

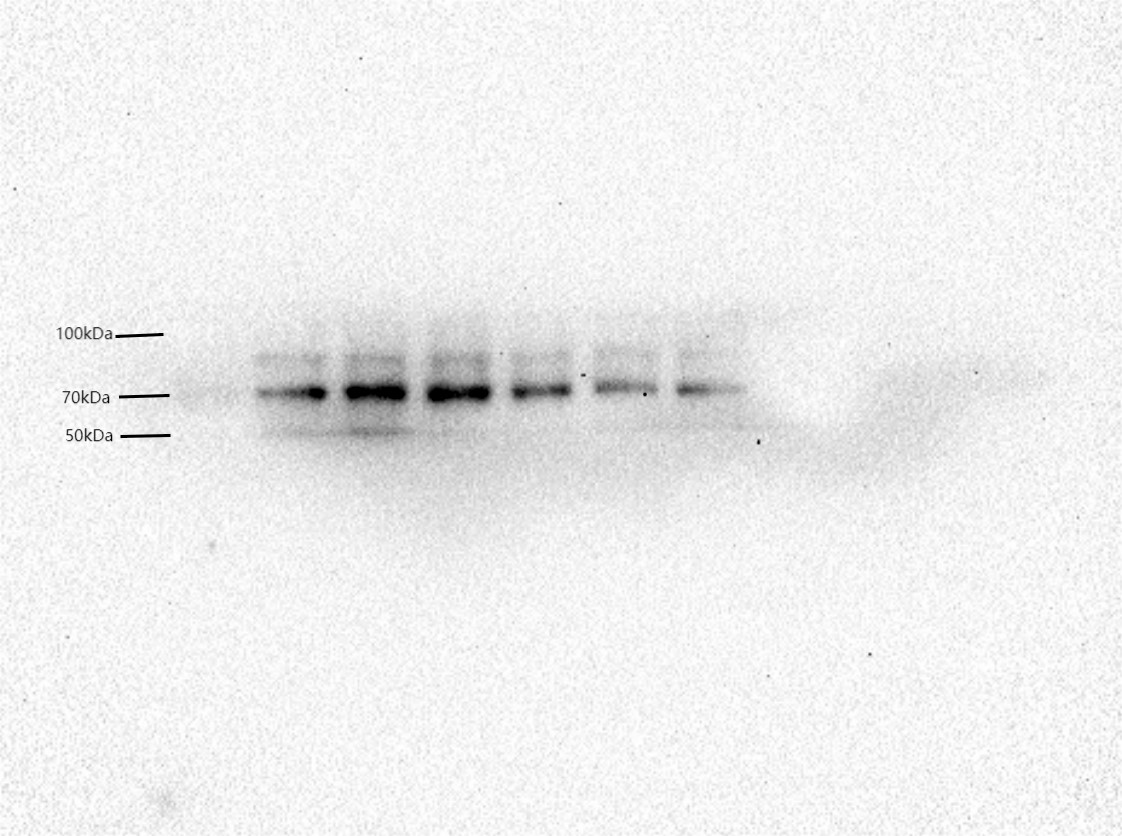

Supplement: Figure 2—source data 9. [file elife-80625-fig2-data9.zip › Figure 2-source data 9/Figure 2-source data I/COX-2/COX-2_A.jpg]

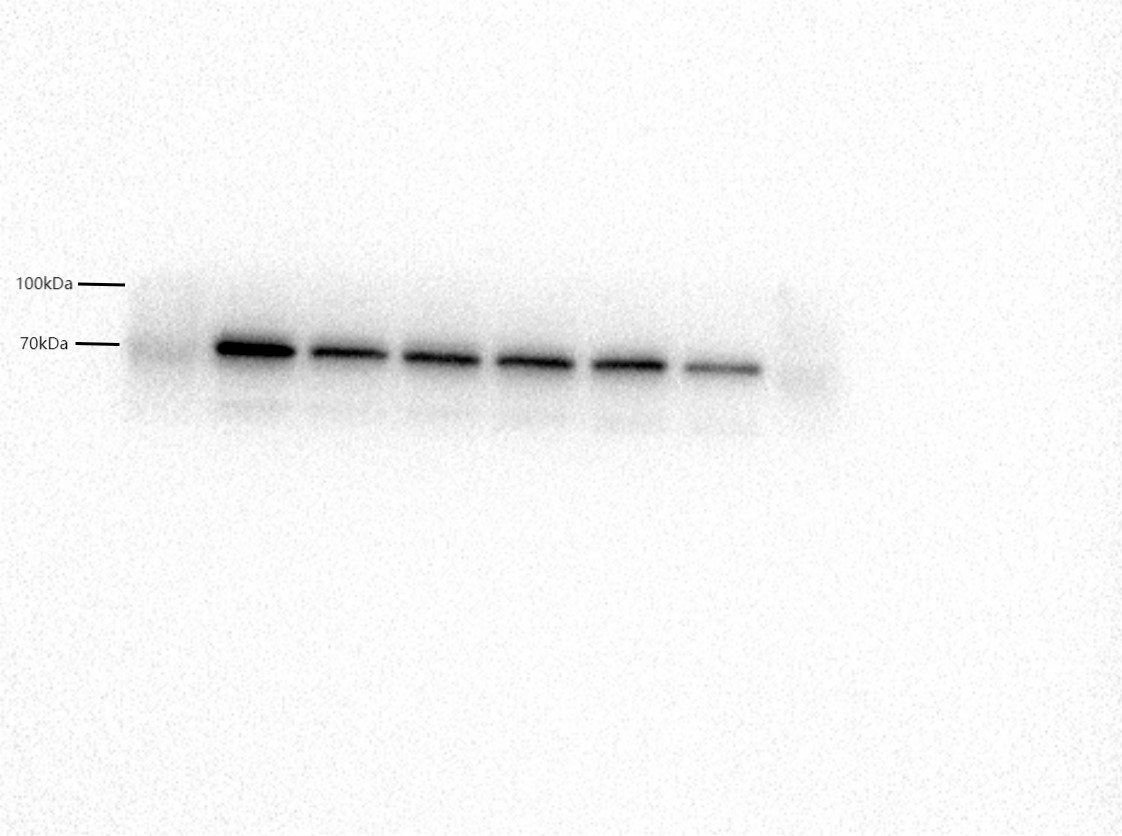

Supplement: Figure 2—source data 9. [file elife-80625-fig2-data9.zip › Figure 2-source data 9/Figure 2-source data I/p65/p65_A.jpg]

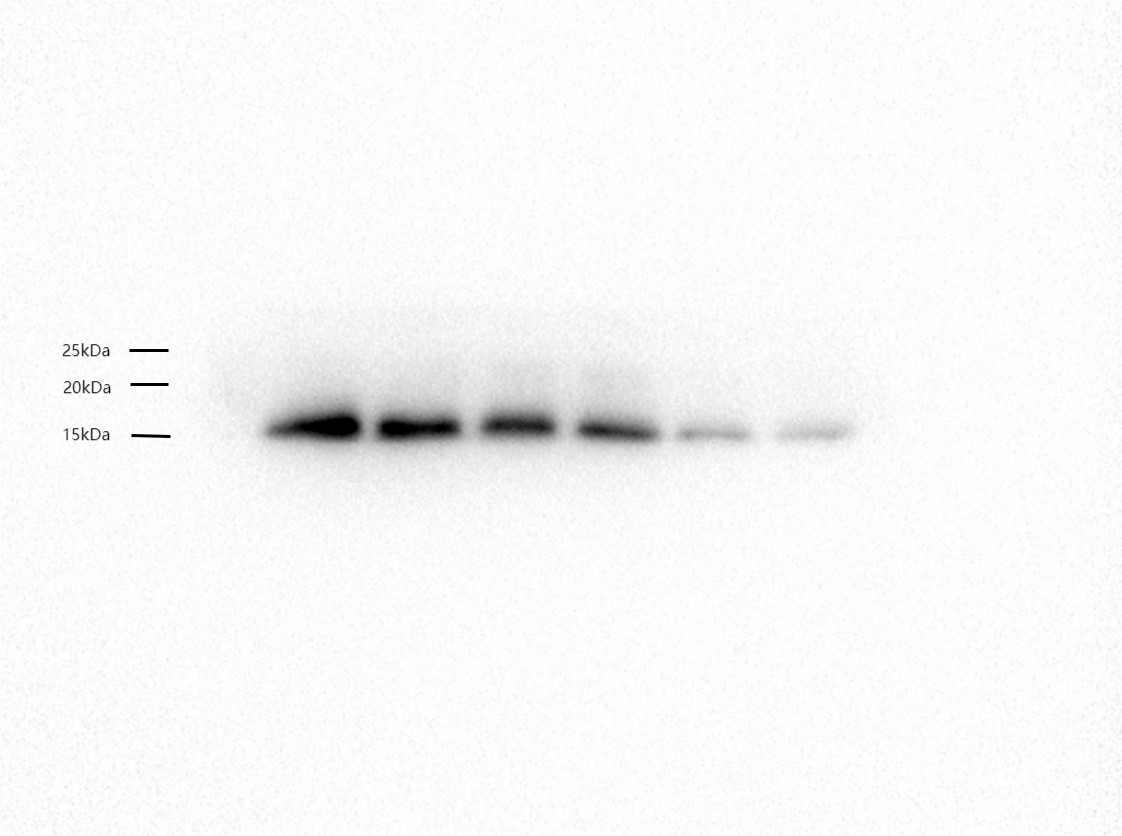

Supplement: Figure 2—source data 9. [file elife-80625-fig2-data9.zip › Figure 2-source data 9/Figure 2-source data I/Survivin/Survivin_A.jpg]

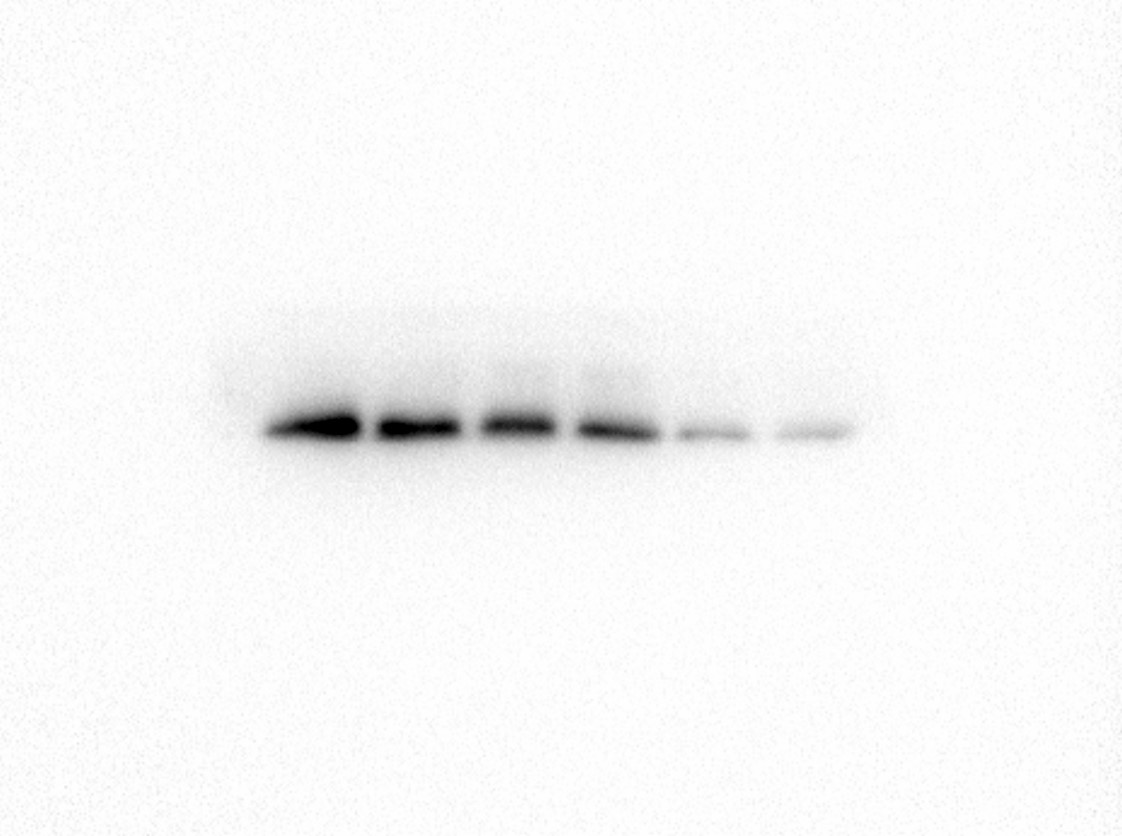

Supplement: Figure 2—source data 9. [file elife-80625-fig2-data9.zip › Figure 2-source data 9/Figure 2-source data I/Survivin/Survivin_B.jpg]

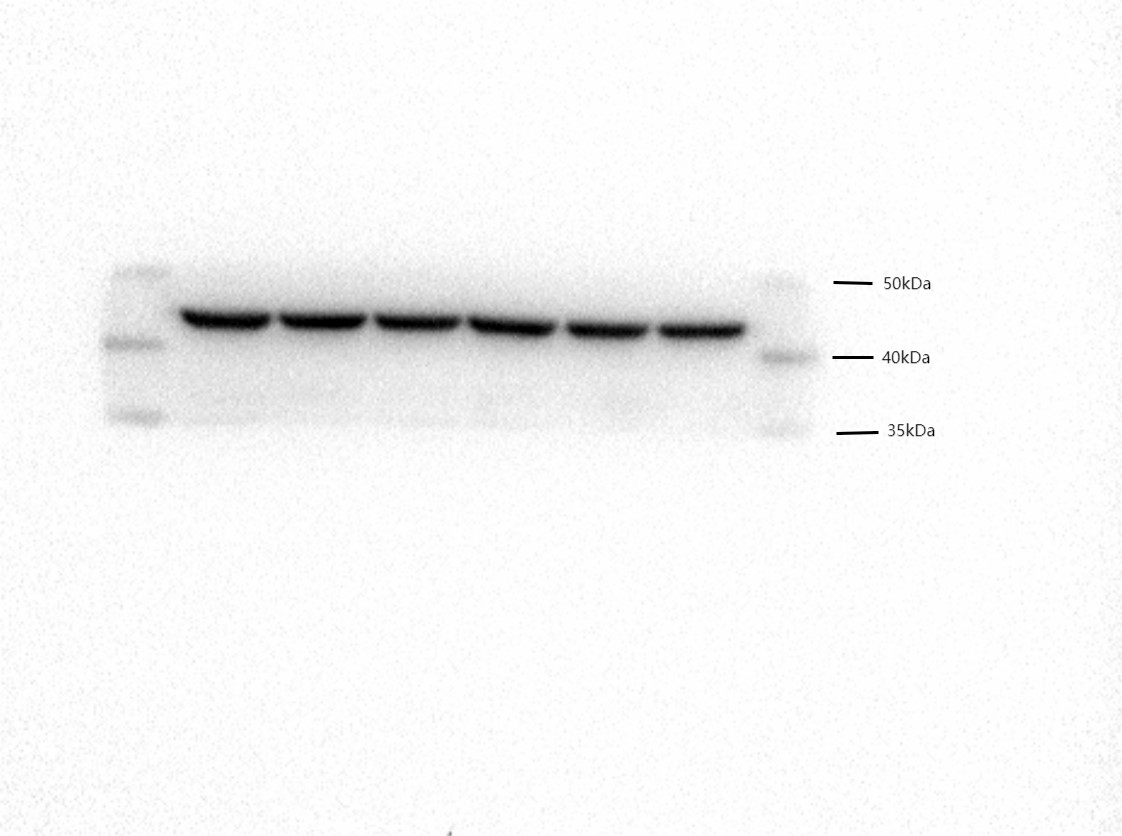

Supplement: Figure 2—source data 9. [file elife-80625-fig2-data9.zip › Figure 2-source data 9/Figure 2-source data I/a┬-actin/a┬-actin_A.jpg]

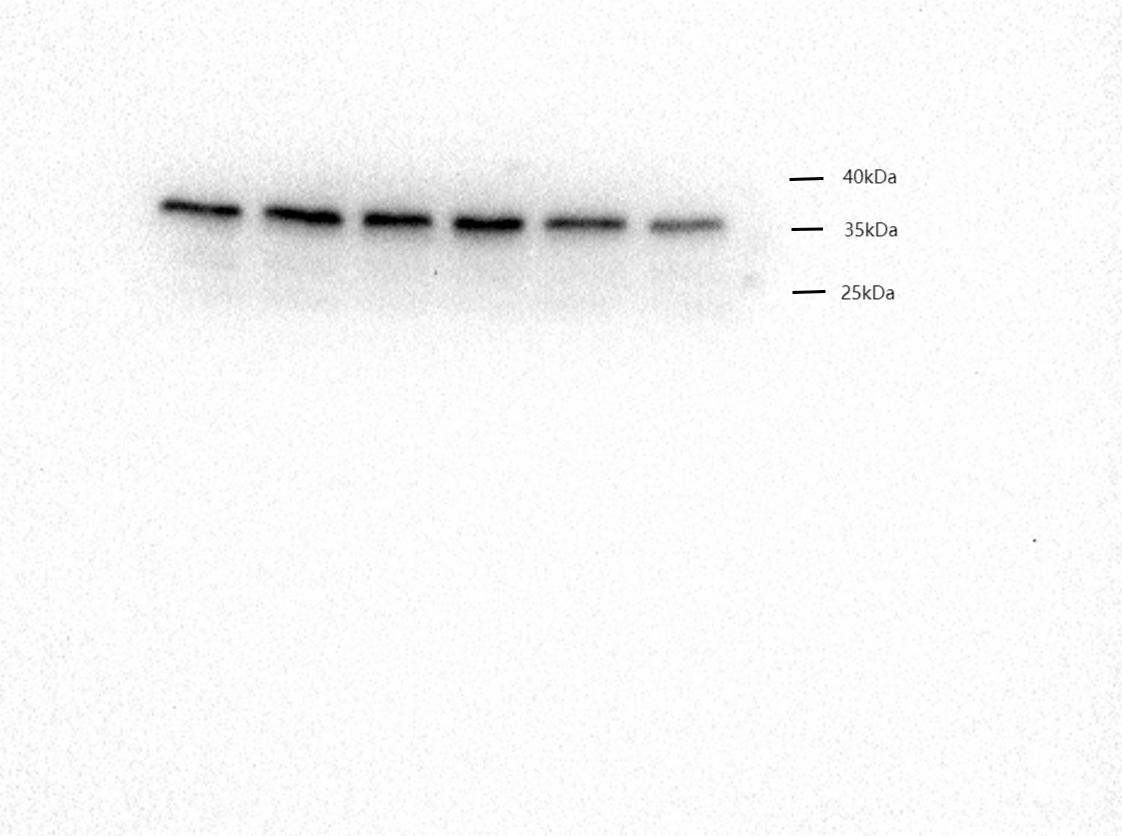

Supplement: Figure 2—source data 10. [file elife-80625-fig2-data10.zip › Figure 2-source data 10/Figure 2-source data J/cytoplasm/Ia╩Ba┴ - A.jpg]

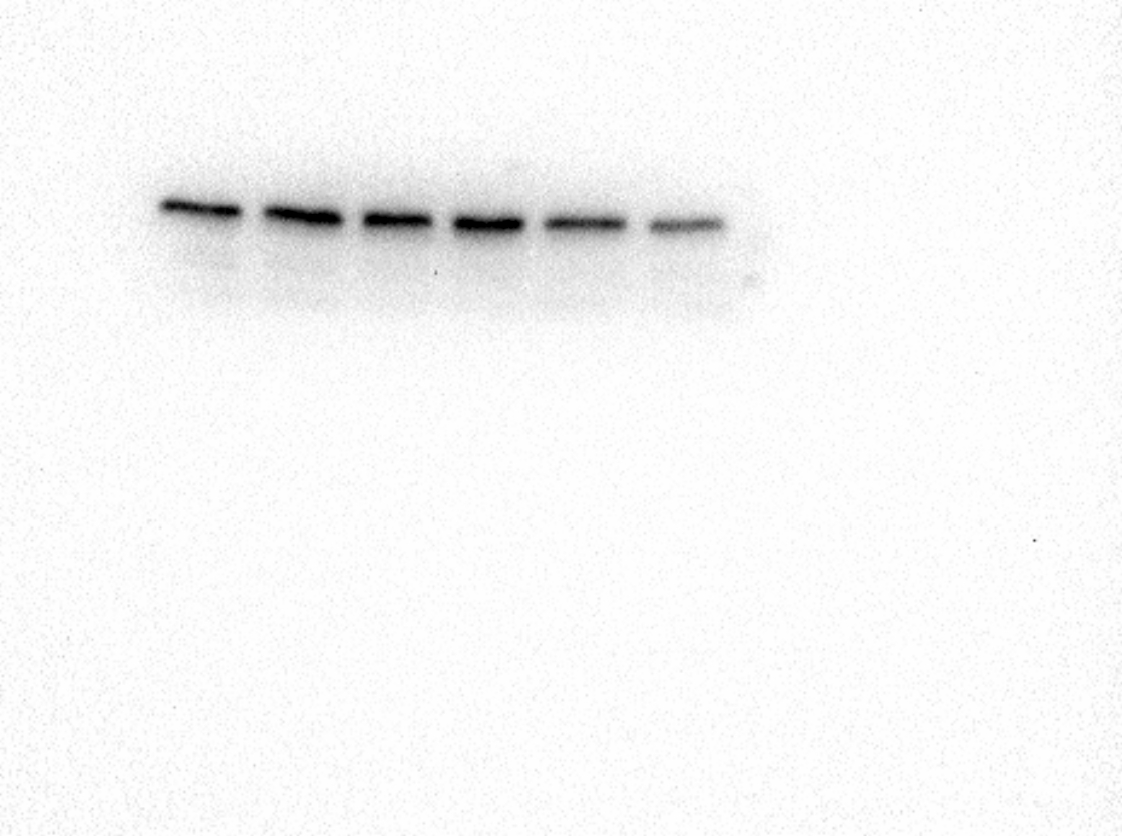

Supplement: Figure 2—source data 10. [file elife-80625-fig2-data10.zip › Figure 2-source data 10/Figure 2-source data J/cytoplasm/Ia╩Ba┴.jpg]

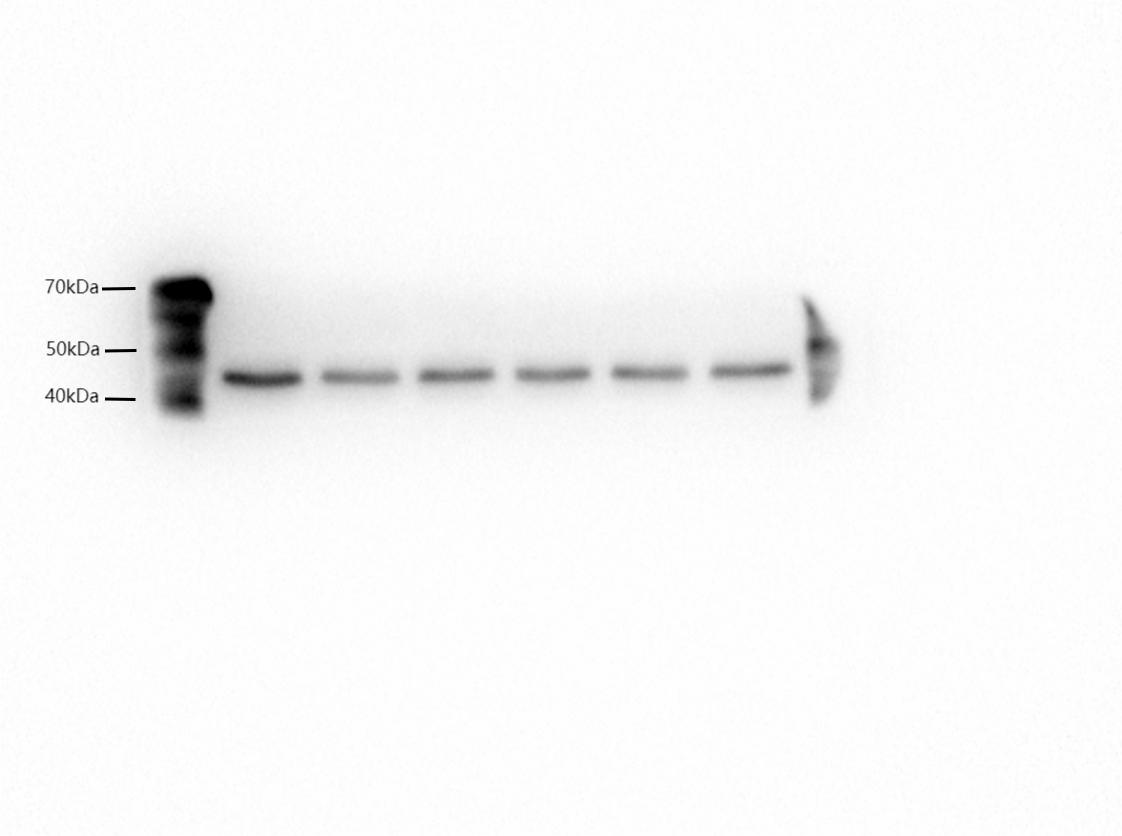

Supplement: Figure 2—source data 10. [file elife-80625-fig2-data10.zip › Figure 2-source data 10/Figure 2-source data J/cytoplasm/a┬-tubulin - A.jpg]

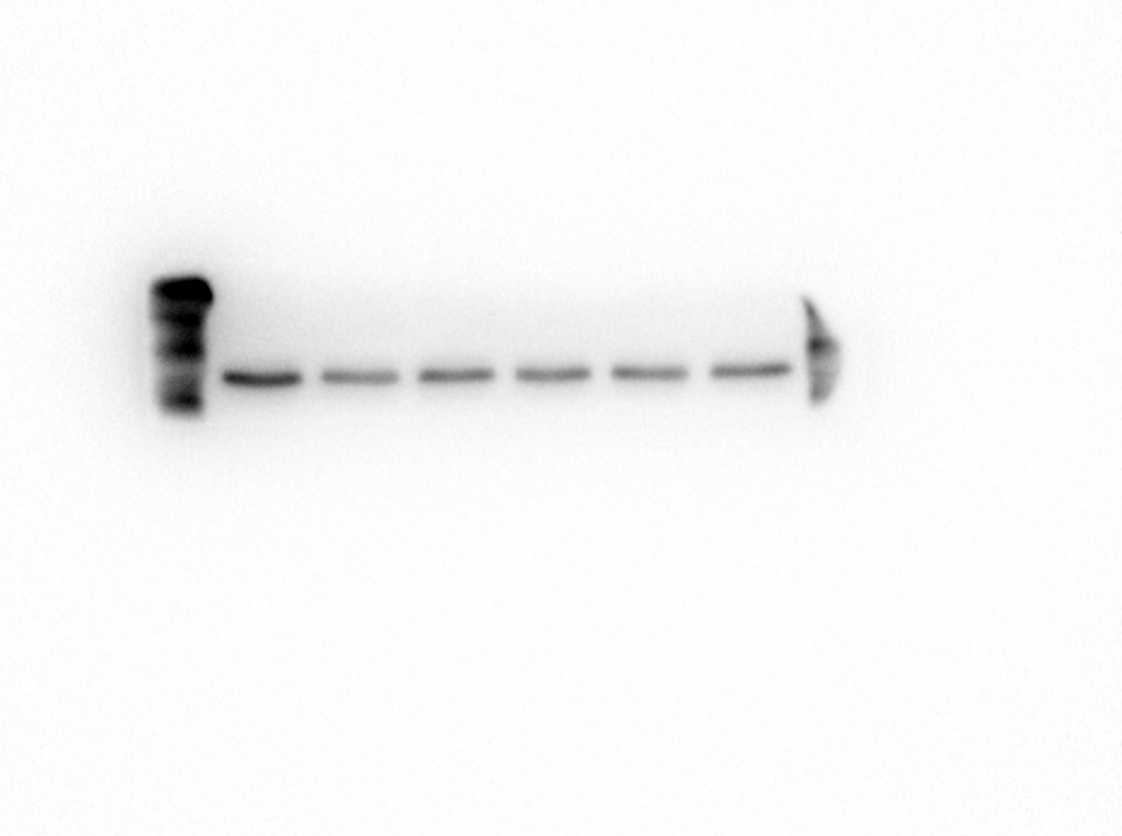

Supplement: Figure 2—source data 10. [file elife-80625-fig2-data10.zip › Figure 2-source data 10/Figure 2-source data J/cytoplasm/a┬-tubulin.jpg]

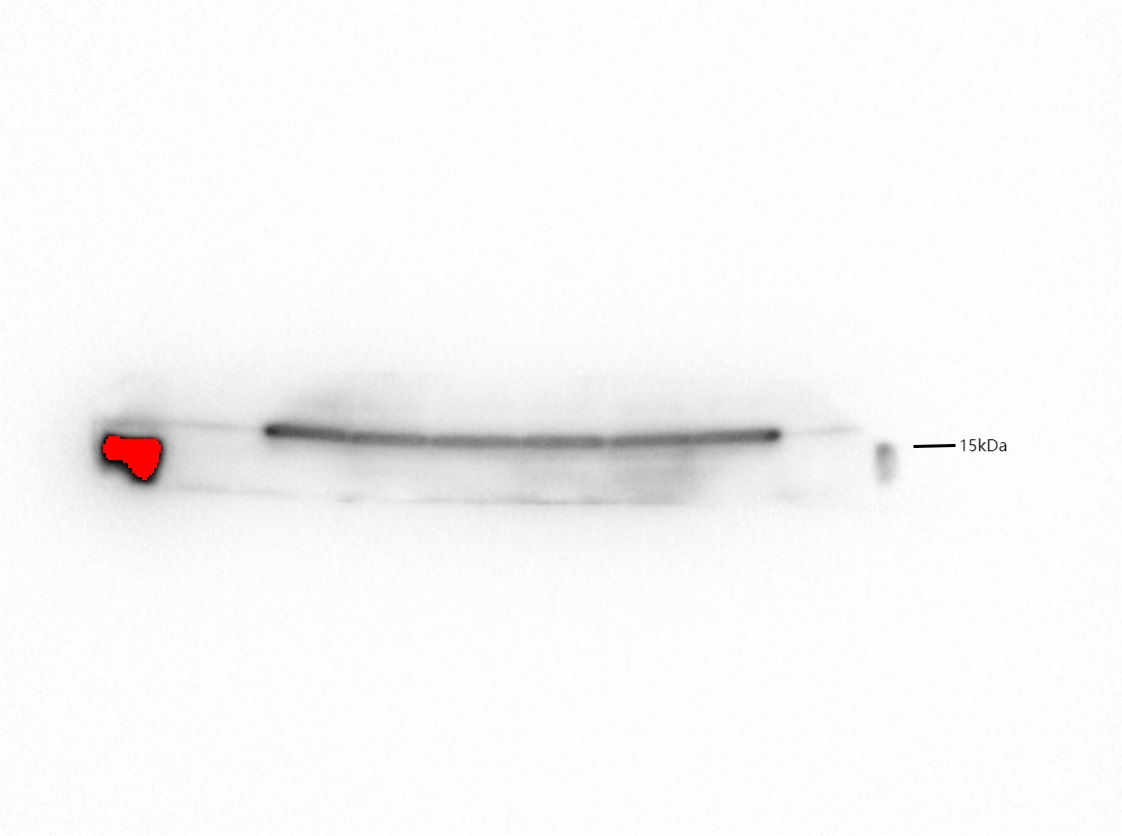

Supplement: Figure 2—source data 10. [file elife-80625-fig2-data10.zip › Figure 2-source data 10/Figure 2-source data J/nuclei/Histone-H3_A.jpg]

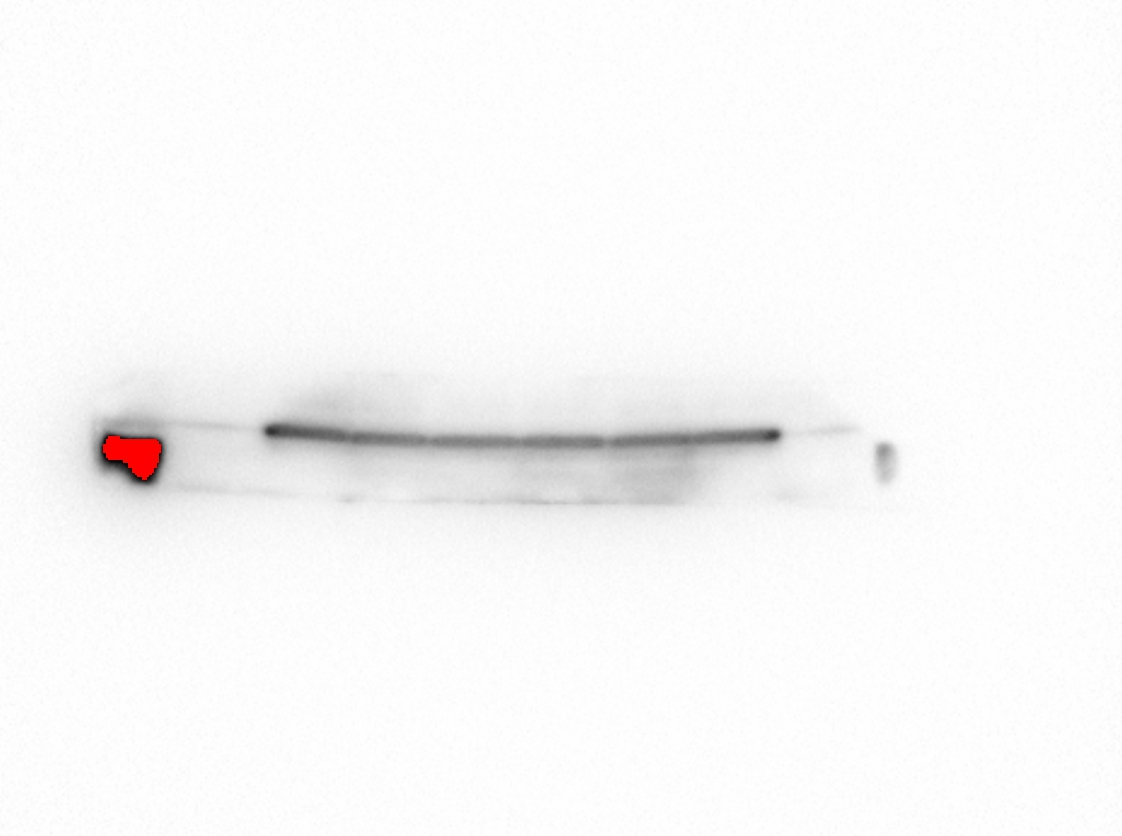

Supplement: Figure 2—source data 10. [file elife-80625-fig2-data10.zip › Figure 2-source data 10/Figure 2-source data J/nuclei/Histone-H3_B.jpg]

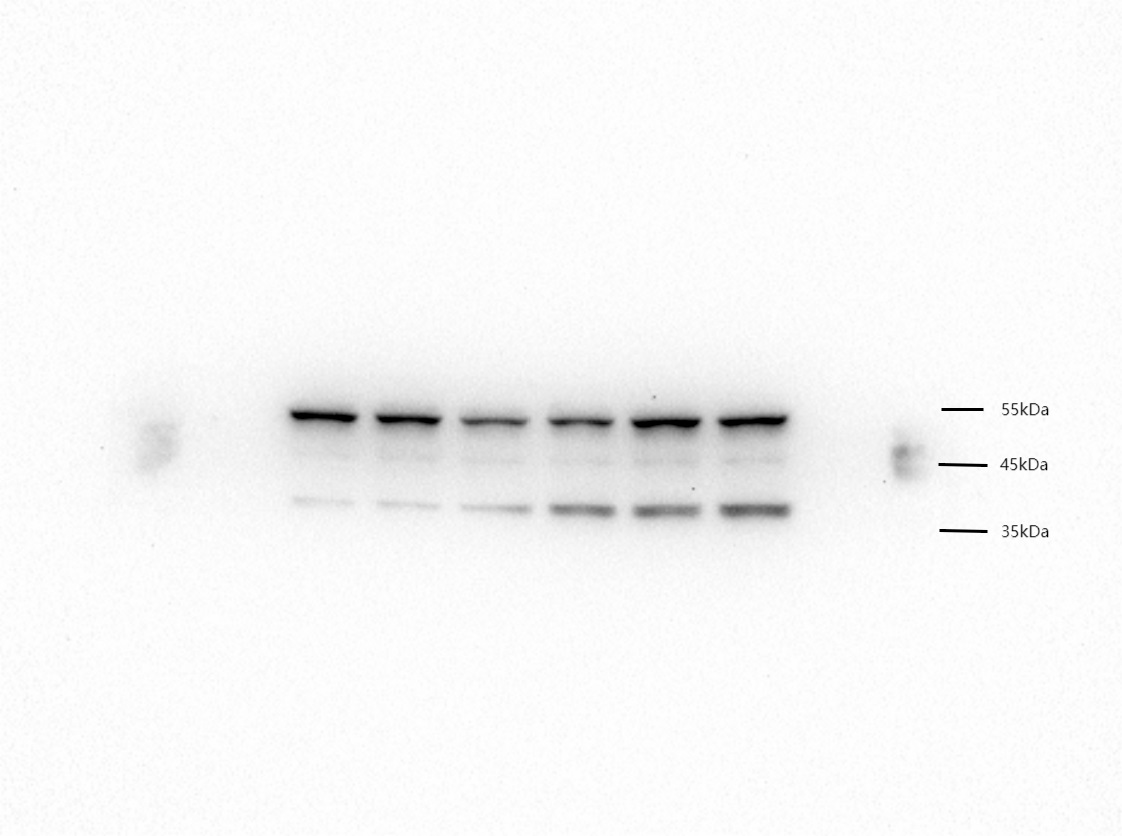

Supplement: Figure 2—source data 10. [file elife-80625-fig2-data10.zip › Figure 2-source data 10/Figure 2-source data J/nuclei/Ia╩B-a┴_A.jpg]

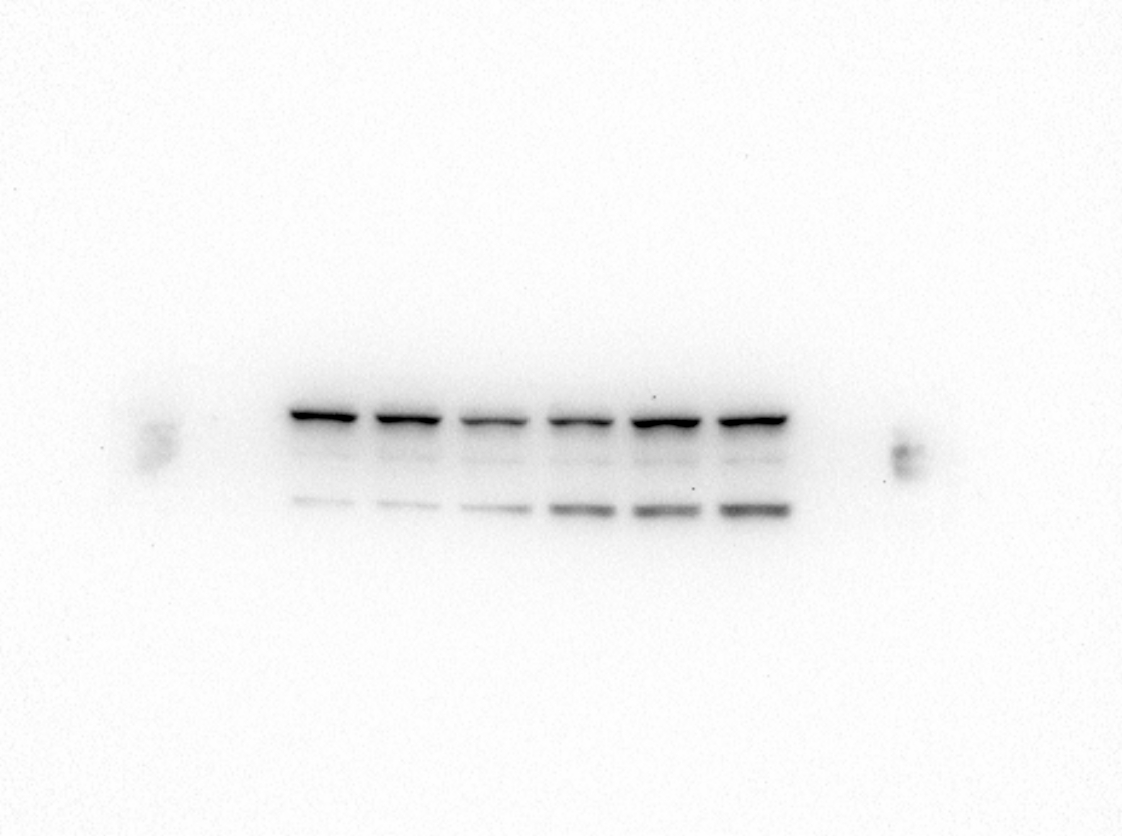

Supplement: Figure 2—source data 10. [file elife-80625-fig2-data10.zip › Figure 2-source data 10/Figure 2-source data J/nuclei/Ia╩B-a┴_B.jpg]

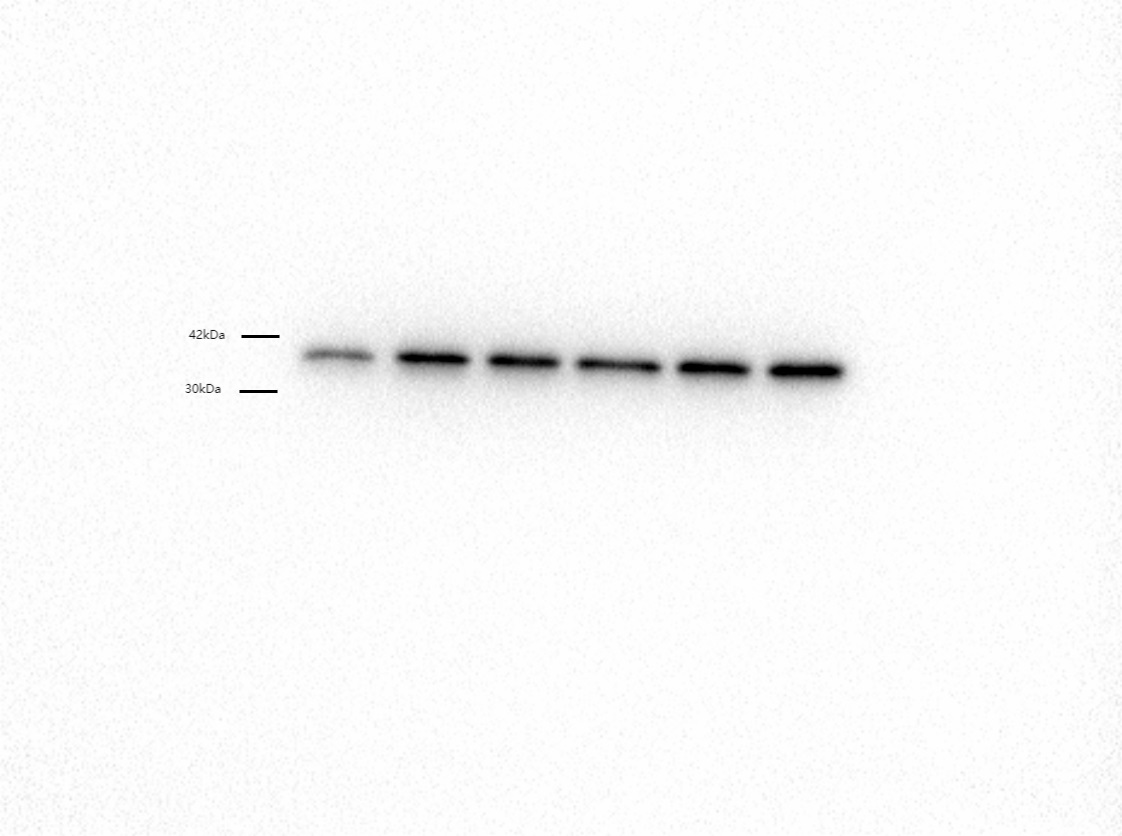

Supplement: Figure 2—source data 10. [file elife-80625-fig2-data10.zip › Figure 2-source data 10/Figure 2-source data J/whole cell protein/Ia╩B-a┴_A.jpg]

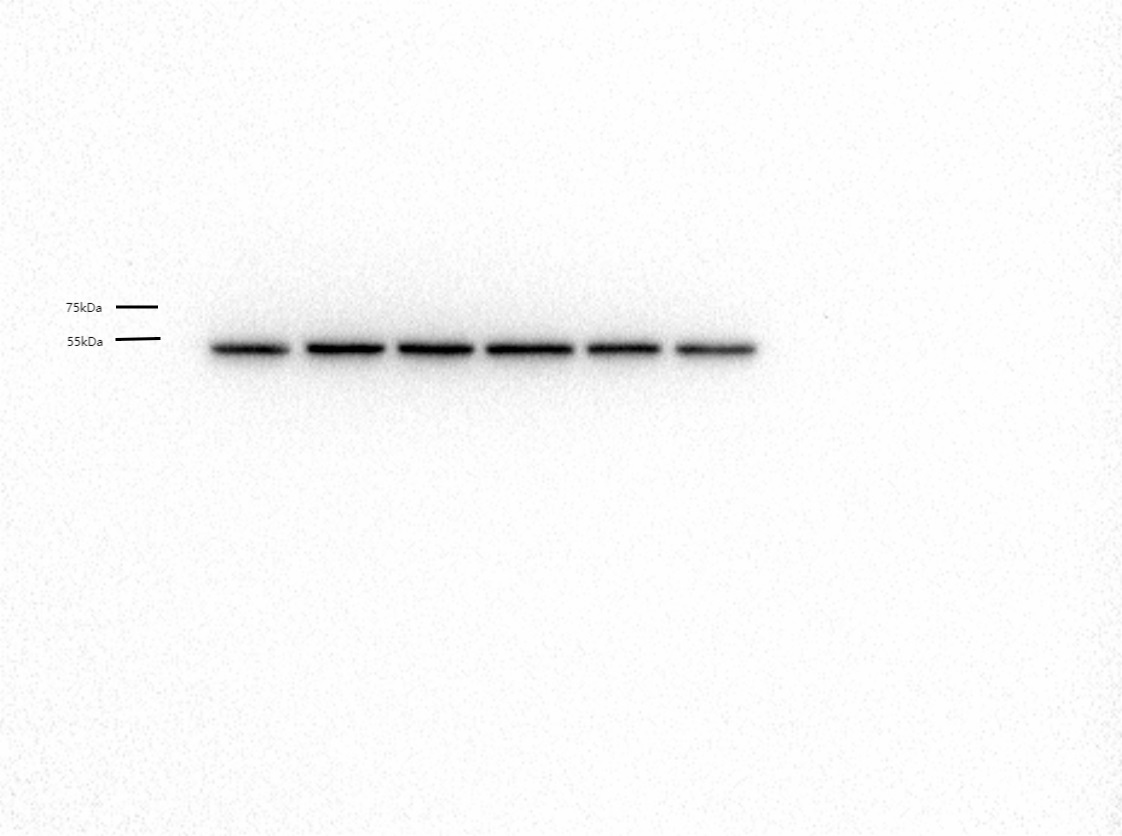

Supplement: Figure 2—source data 10. [file elife-80625-fig2-data10.zip › Figure 2-source data 10/Figure 2-source data J/whole cell protein/a┬-tubulin_A.jpg]

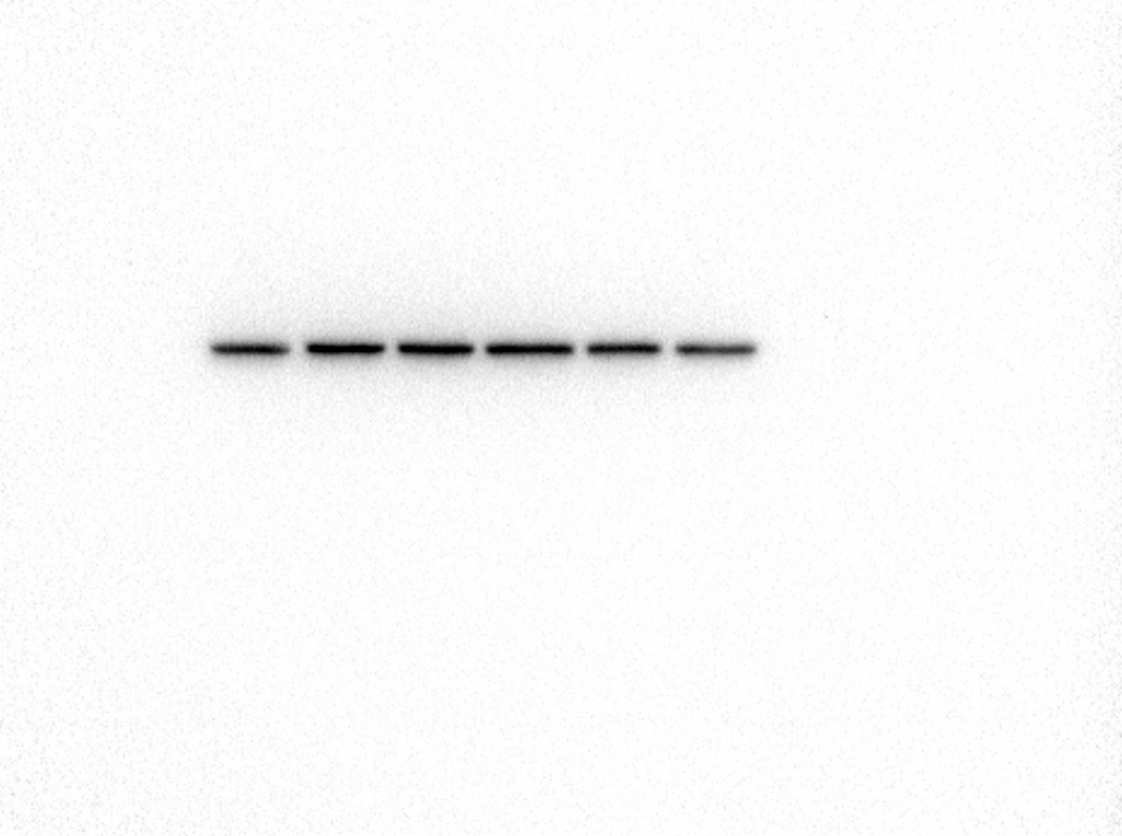

Supplement: Figure 2—source data 10. [file elife-80625-fig2-data10.zip › Figure 2-source data 10/Figure 2-source data J/whole cell protein/a┬-tubulin_B.jpg]

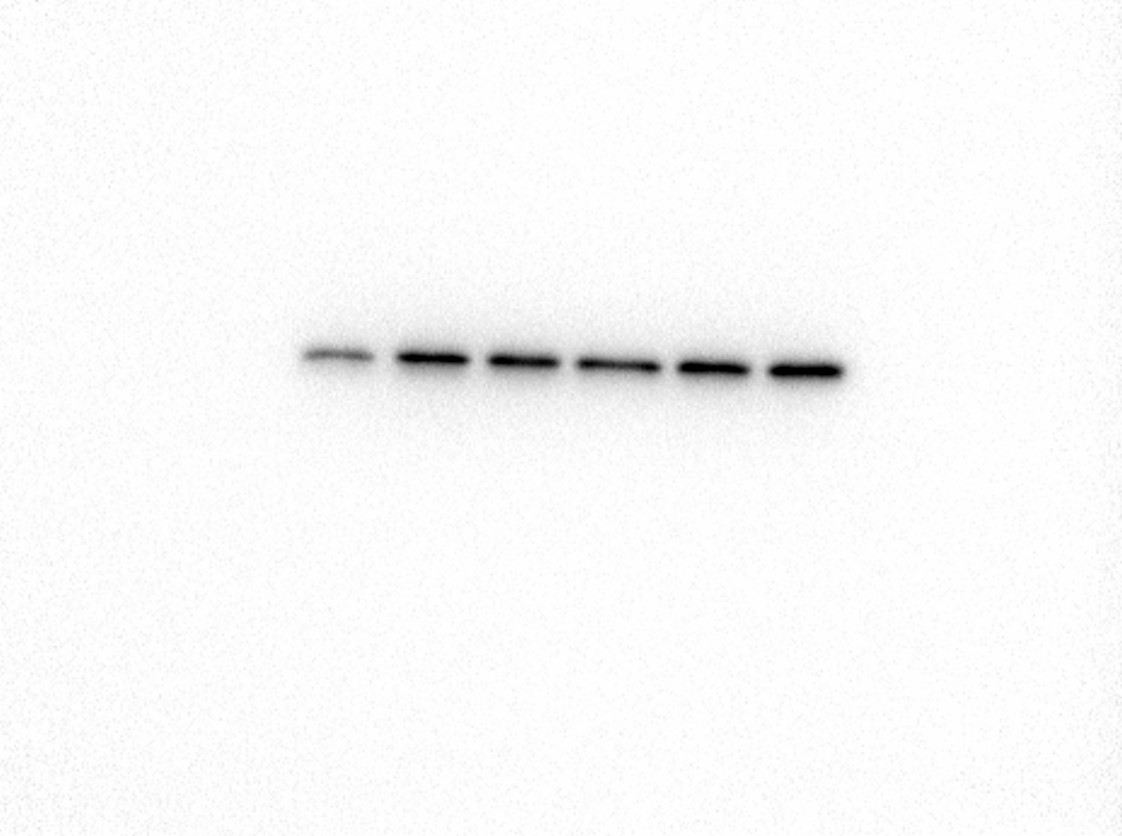

Supplement: Figure 2—source data 10. [file elife-80625-fig2-data10.zip › Figure 2-source data 10/Figure 2-source data J/whole cell protein/Ia╩B-a┴_B.jpg]

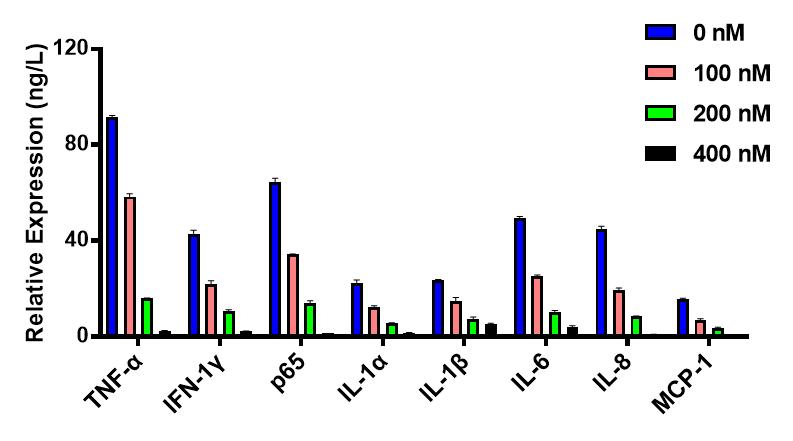

Supplement: Figure 2—source data 11. [file elife-80625-fig2-data11.zip › Figure 2-source data 11/K.jpg]

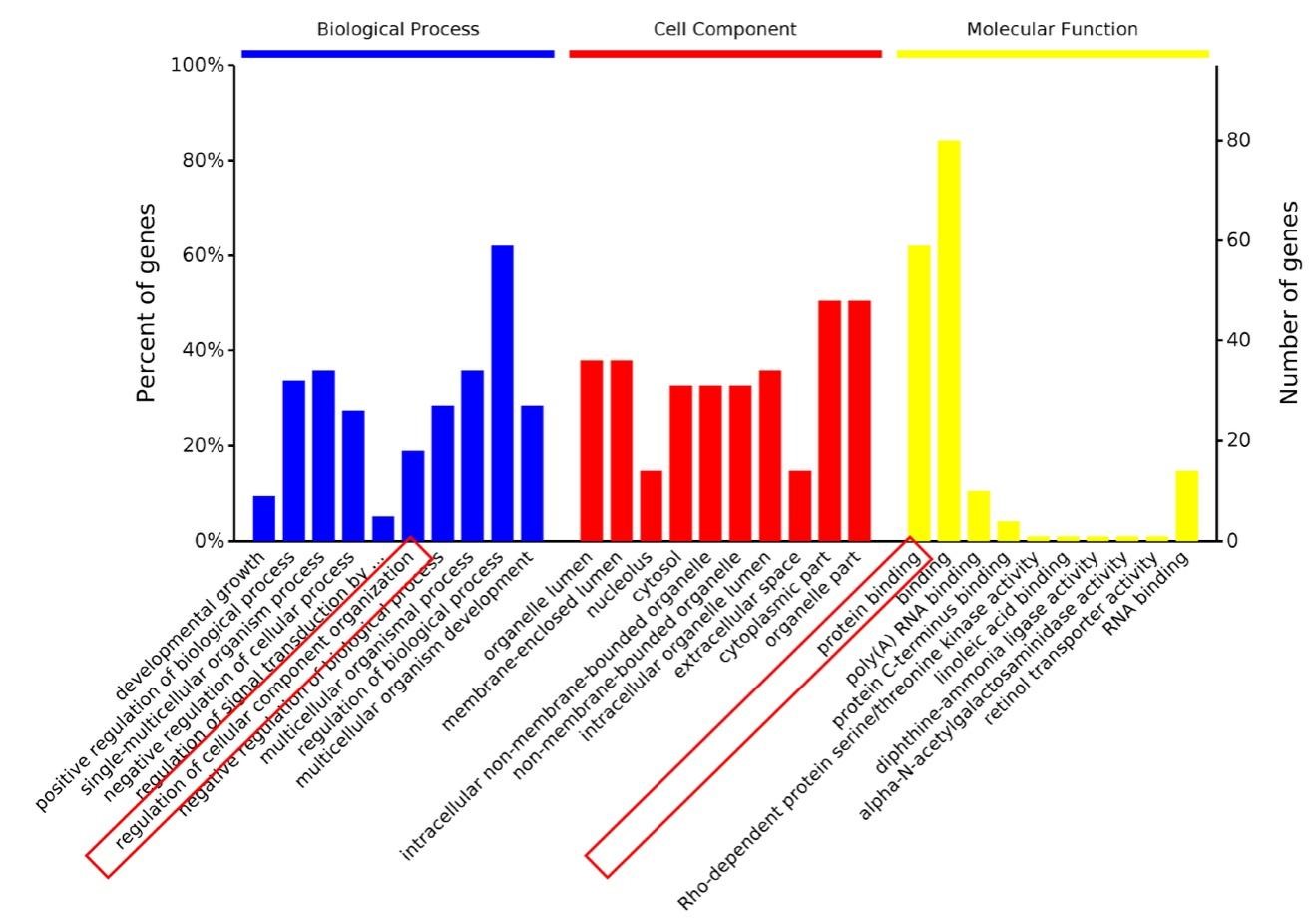

Supplement: Figure 2—figure supplement 1—source data 1. [file elife-80625-fig2-figsupp1-data1.zip › Figure 2-figure supplement 1-source data/figure 2-figure supplement 1.jpg]

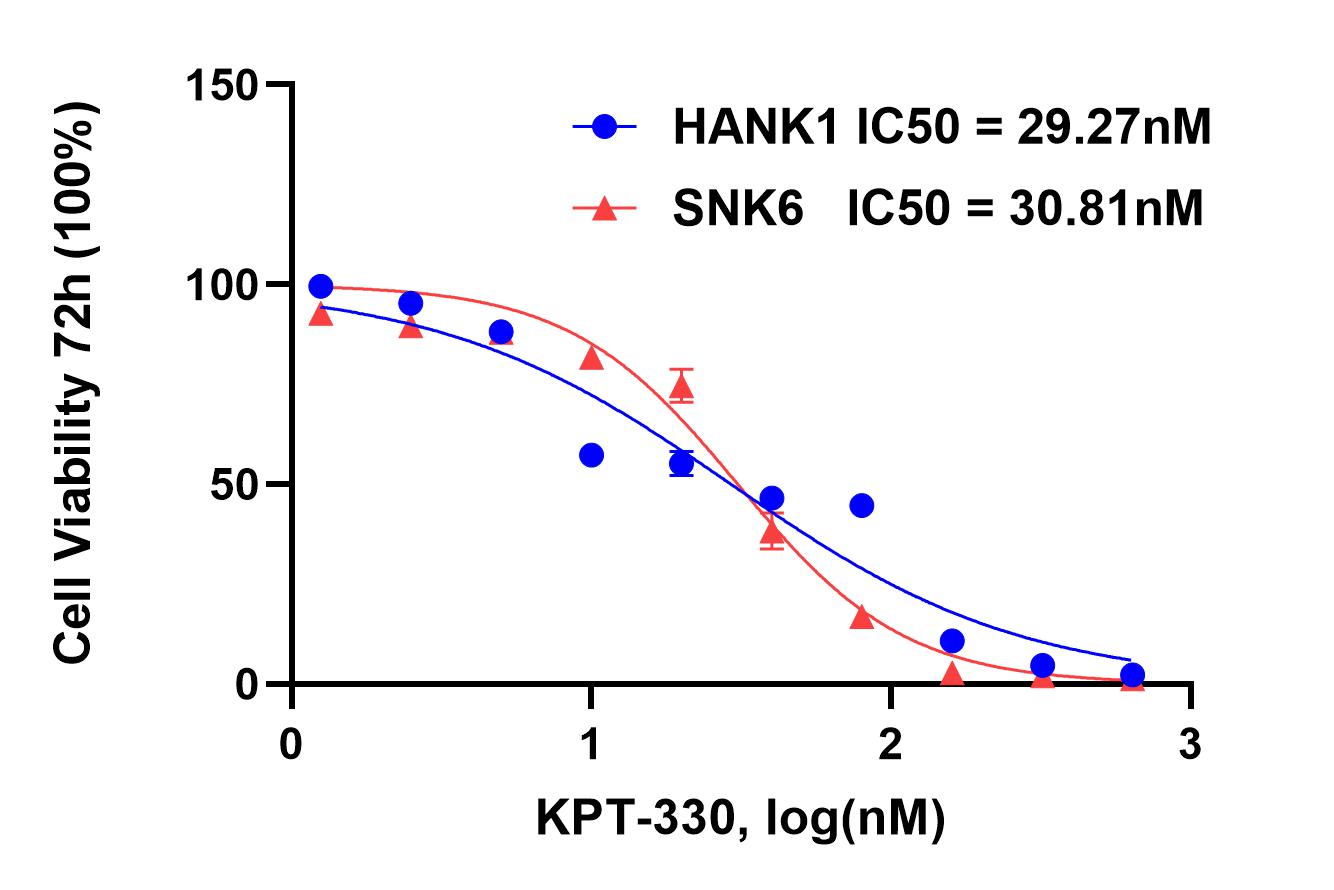

Supplement: Figure 2—figure supplement 2—source data 1. [file elife-80625-fig2-figsupp2-data1.zip › Figure 2 - figure supplement 2 - source data/Figure 2 - figure supplement 2.jpg]

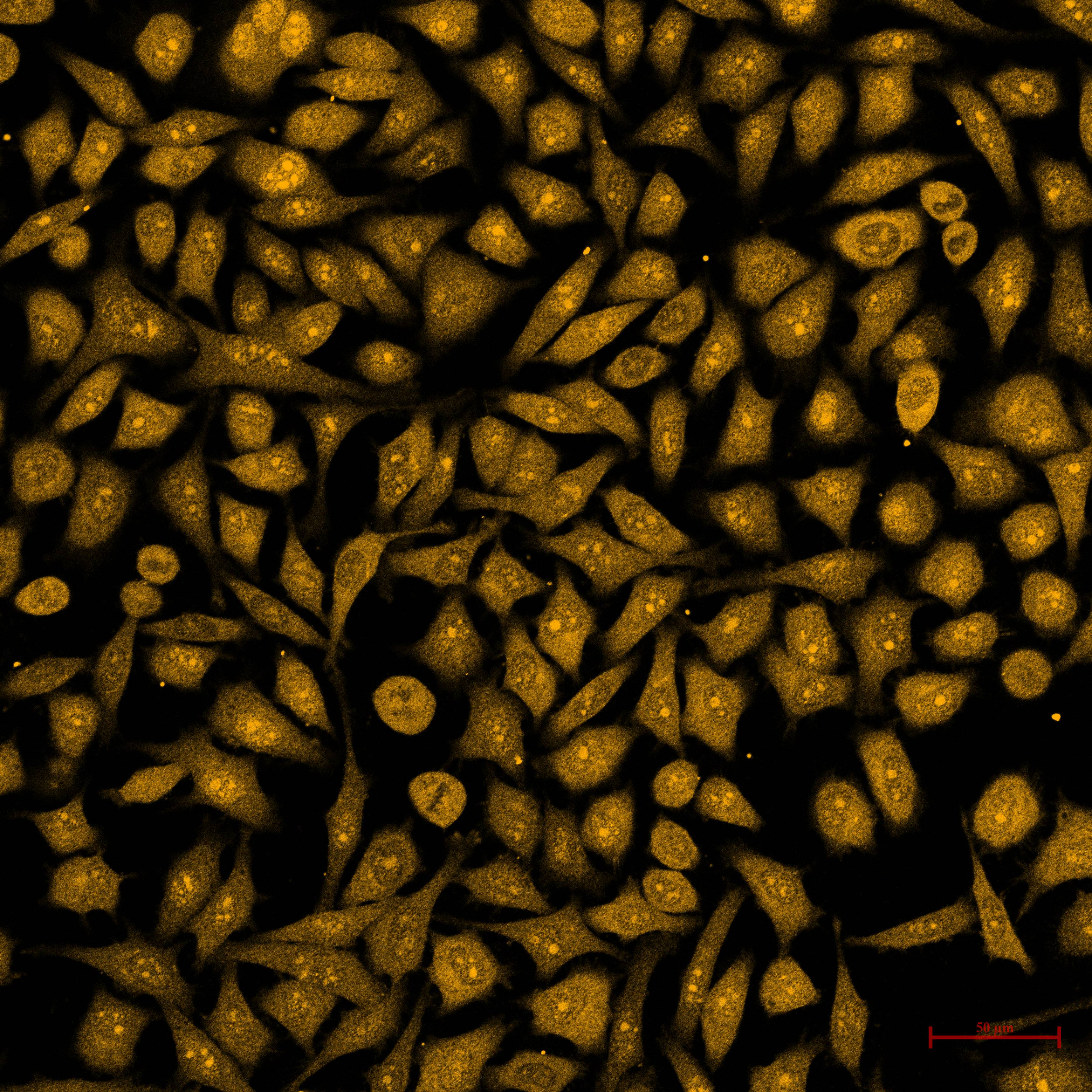

Supplement: Figure 2—figure supplement 3—source data 1. — The medium (containing LFS-1107) was removed and replaced with new medium in the Wash group. Fixed cells were stained for IκBα (orange) and DAPI (blue). [file elife-80625-fig2-figsupp3-data1.zip › Figure 2-figure supplement 3-source data/DMSO/Ia╩Ba┴(1).jpg]

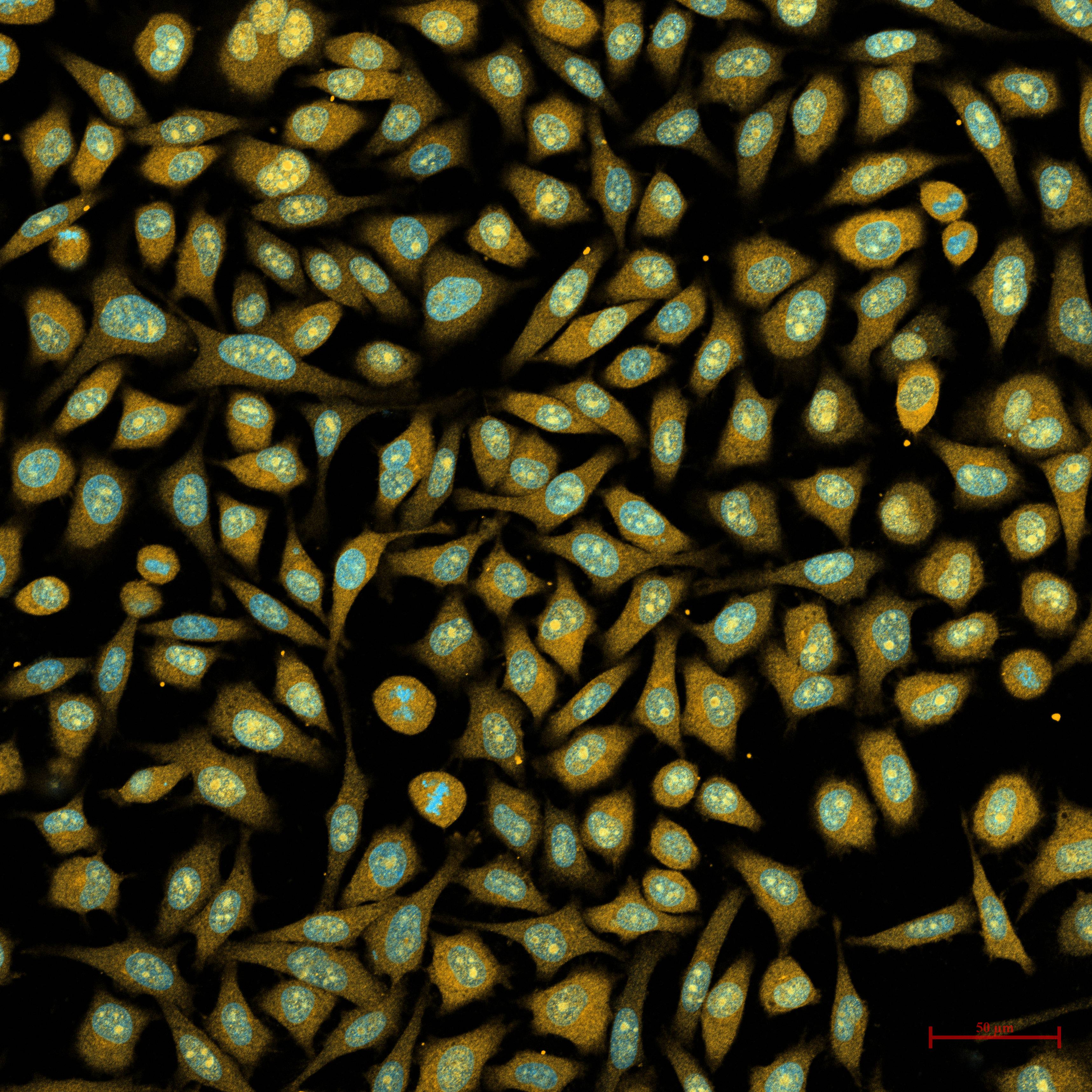

Supplement: Figure 2—figure supplement 3—source data 1. — The medium (containing LFS-1107) was removed and replaced with new medium in the Wash group. Fixed cells were stained for IκBα (orange) and DAPI (blue). [file elife-80625-fig2-figsupp3-data1.zip › Figure 2-figure supplement 3-source data/DMSO/merge(1).jpg]

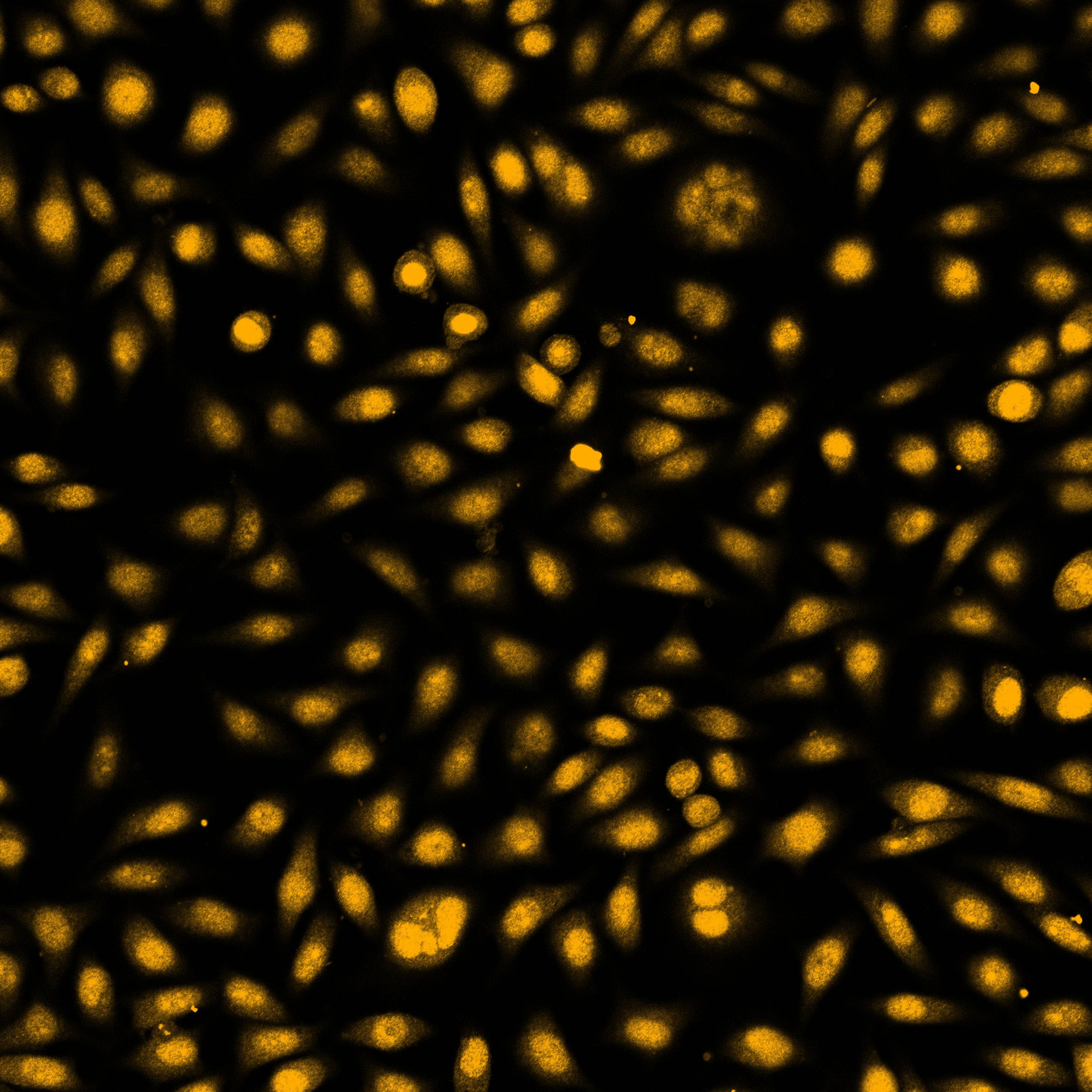

Supplement: Figure 2—figure supplement 3—source data 1. — The medium (containing LFS-1107) was removed and replaced with new medium in the Wash group. Fixed cells were stained for IκBα (orange) and DAPI (blue). [file elife-80625-fig2-figsupp3-data1.zip › Figure 2-figure supplement 3-source data/LFS-1107/Ia╩Ba┴(1).jpg]

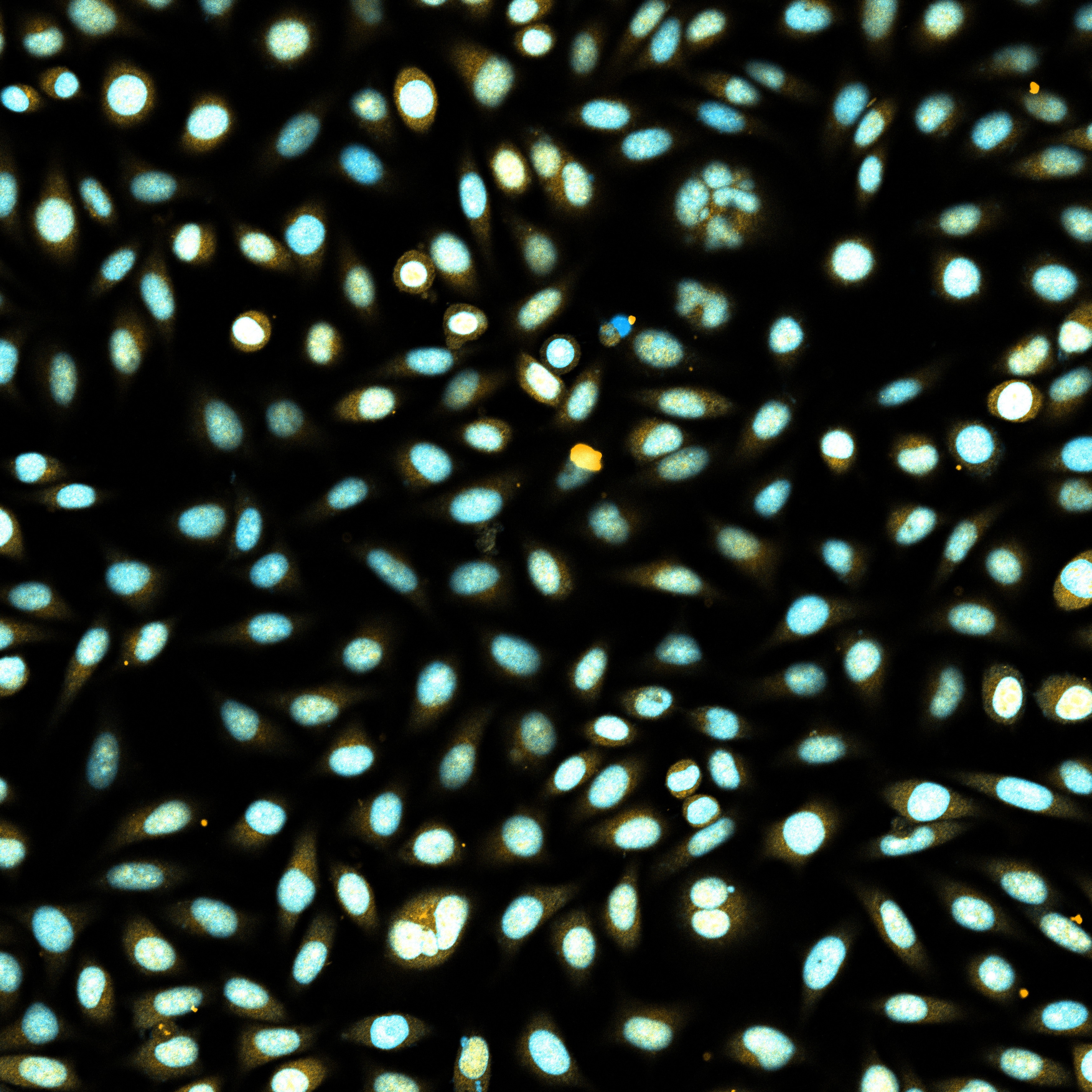

Supplement: Figure 2—figure supplement 3—source data 1. — The medium (containing LFS-1107) was removed and replaced with new medium in the Wash group. Fixed cells were stained for IκBα (orange) and DAPI (blue). [file elife-80625-fig2-figsupp3-data1.zip › Figure 2-figure supplement 3-source data/LFS-1107/Merge.jpg]

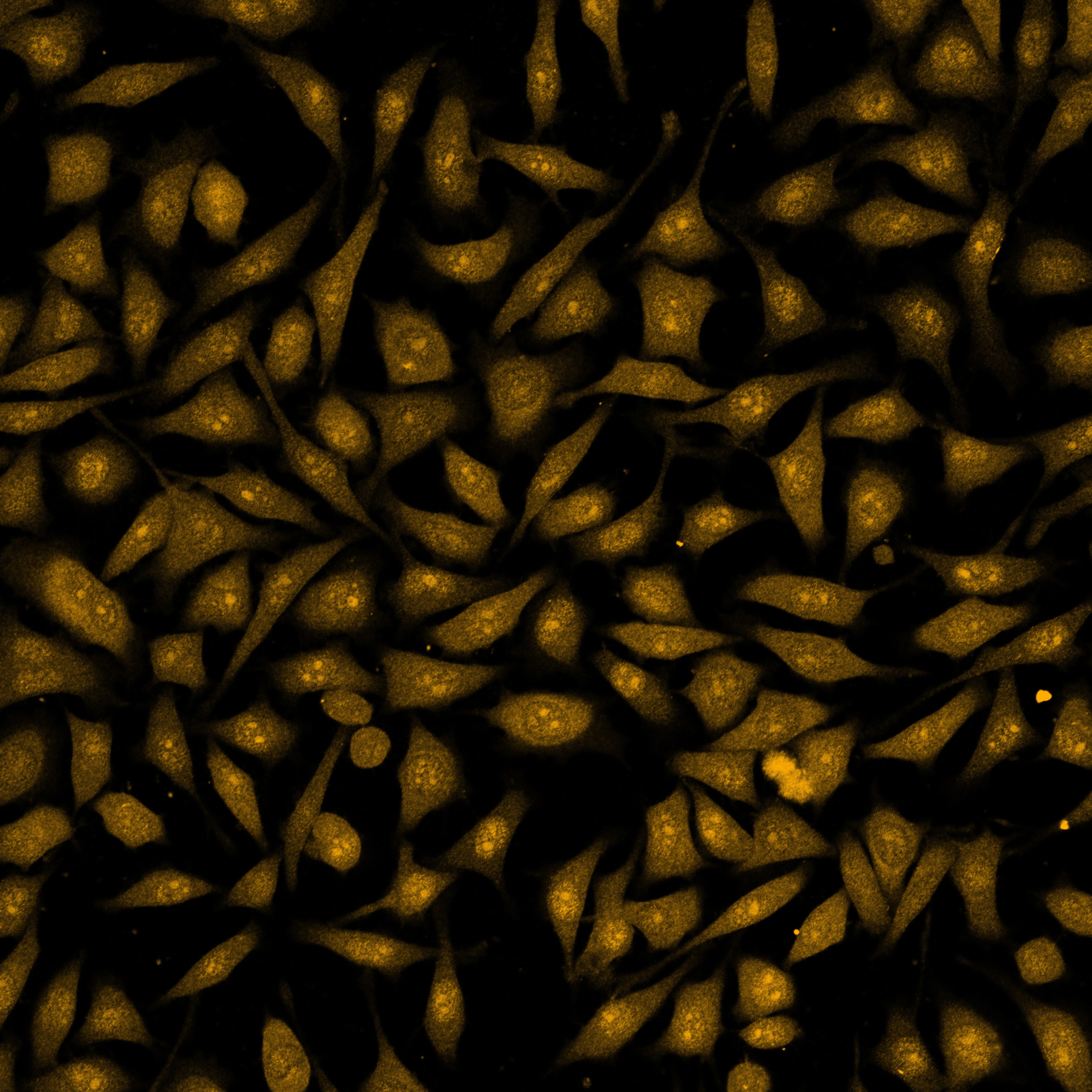

Supplement: Figure 2—figure supplement 3—source data 1. — The medium (containing LFS-1107) was removed and replaced with new medium in the Wash group. Fixed cells were stained for IκBα (orange) and DAPI (blue). [file elife-80625-fig2-figsupp3-data1.zip › Figure 2-figure supplement 3-source data/Wash/Ia╩Ba┴.jpg]

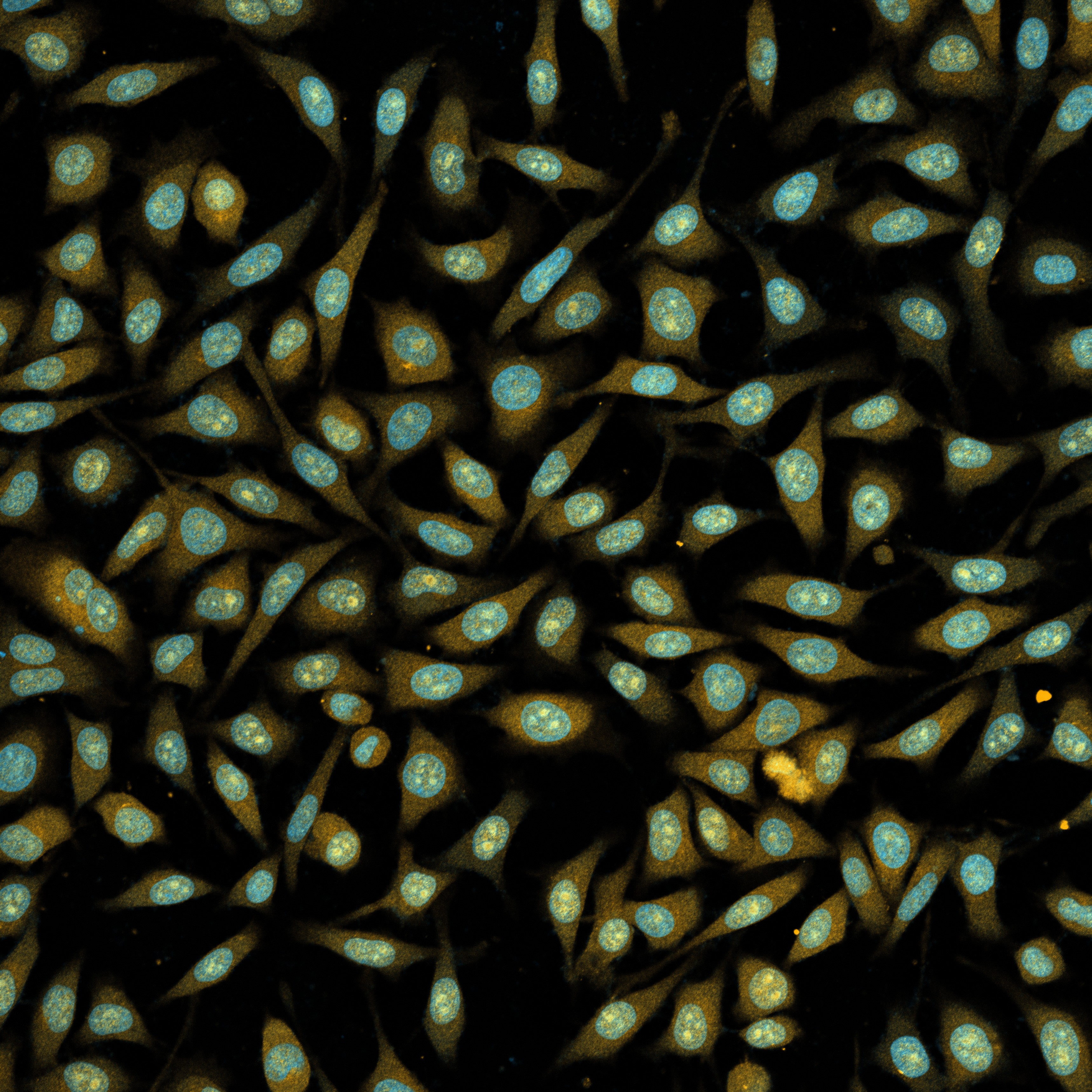

Supplement: Figure 2—figure supplement 3—source data 1. — The medium (containing LFS-1107) was removed and replaced with new medium in the Wash group. Fixed cells were stained for IκBα (orange) and DAPI (blue). [file elife-80625-fig2-figsupp3-data1.zip › Figure 2-figure supplement 3-source data/Wash/Merge.jpg]

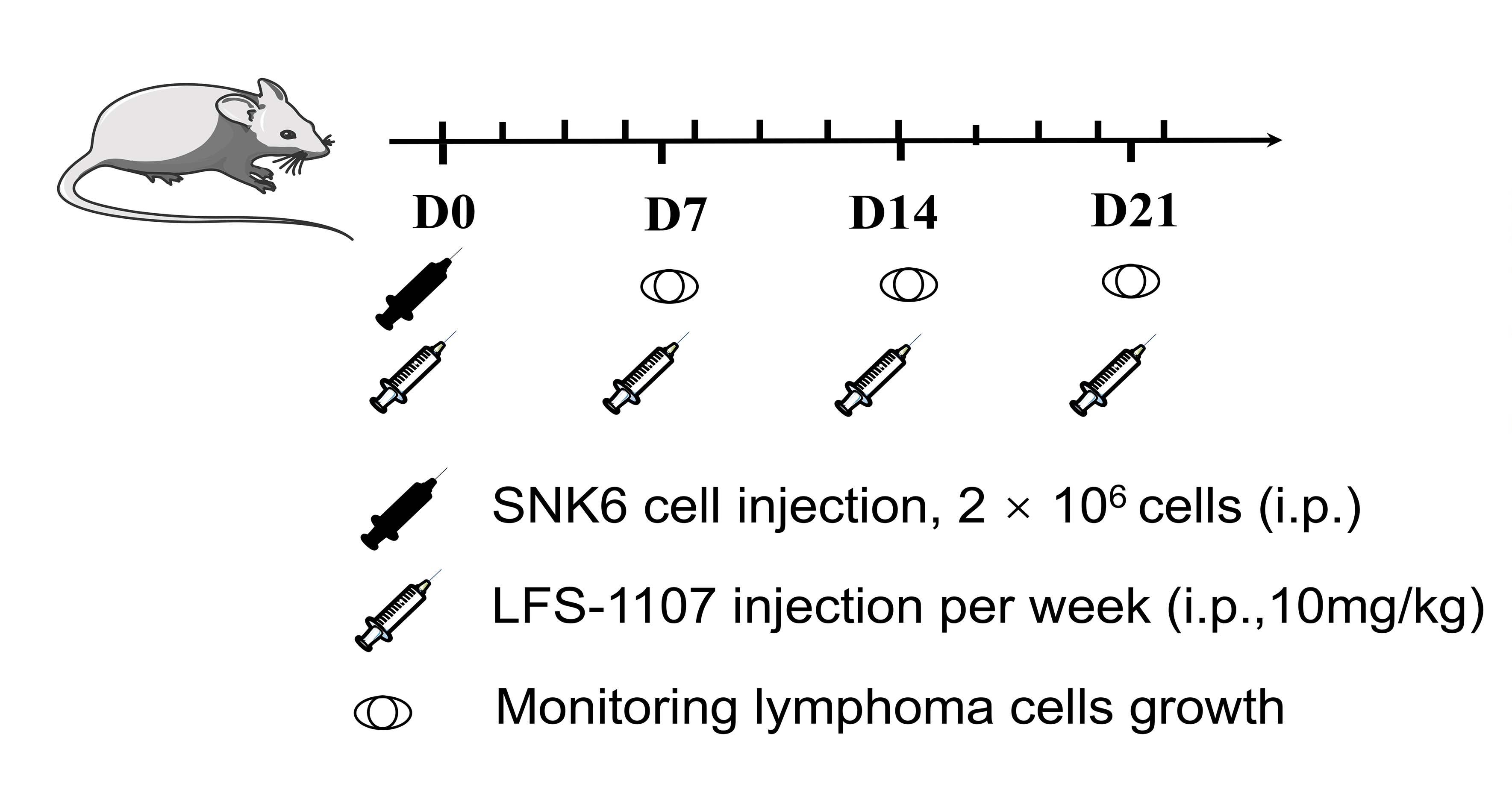

Supplement: Figure 3—source data 1. [file elife-80625-fig3-data1.zip › Figure 3-source data A/Figure-3A.jpg]

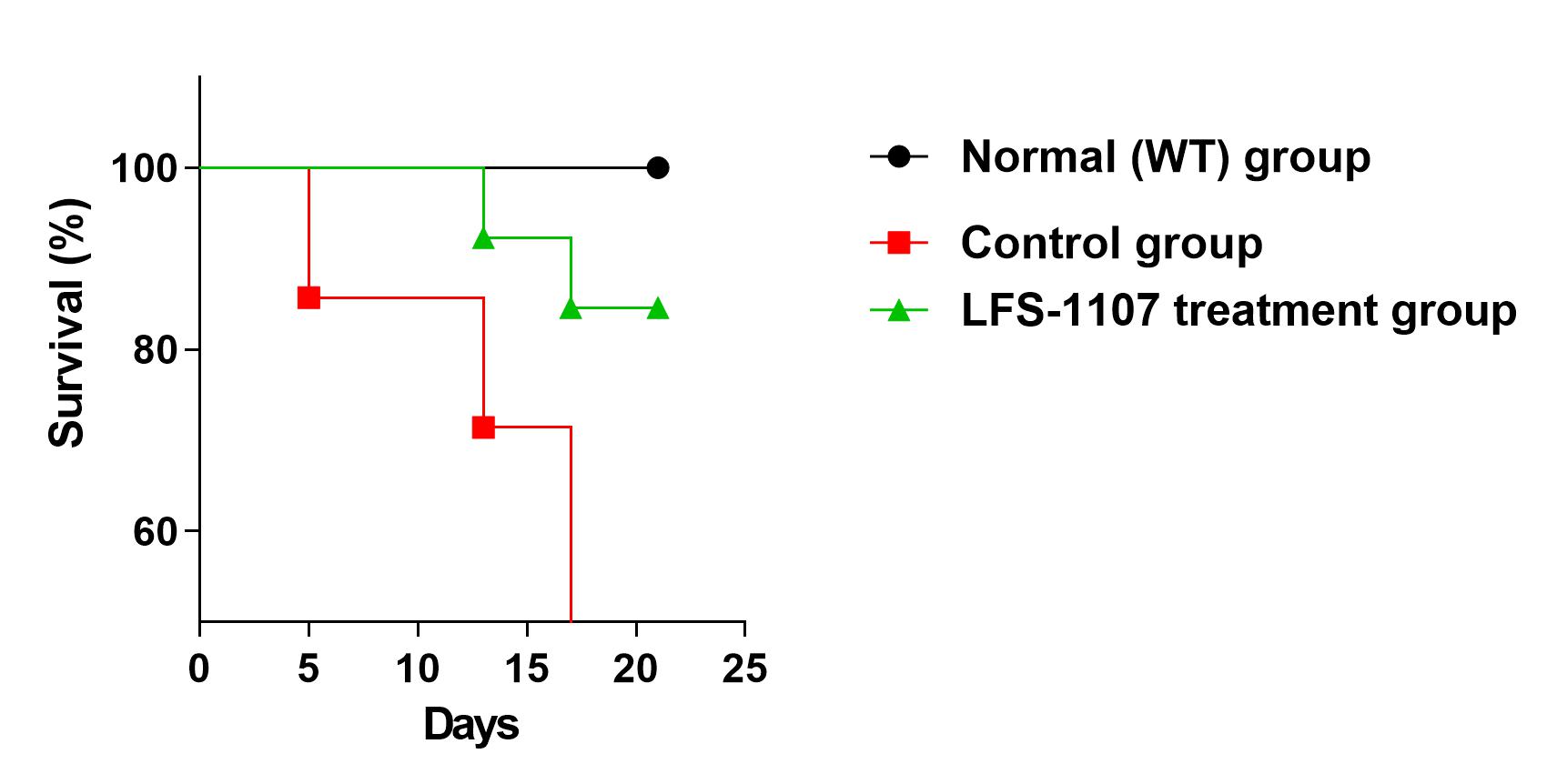

Supplement: Figure 3—source data 2. [file elife-80625-fig3-data2.zip › Figure 3-source data 2/3B.jpg]

**C**

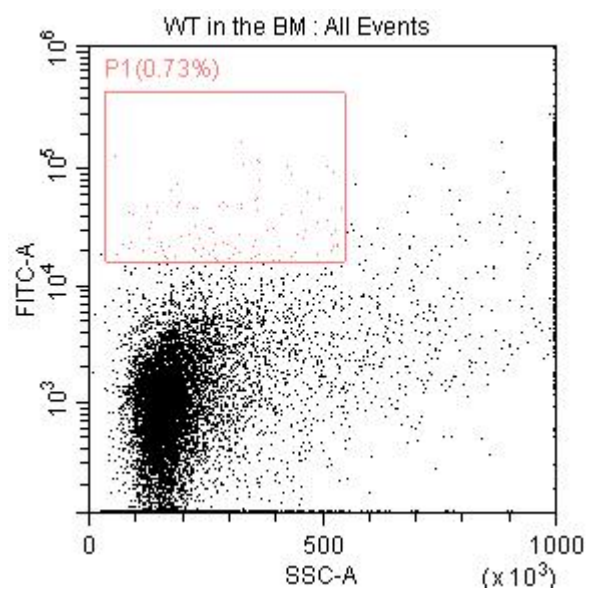

**D**

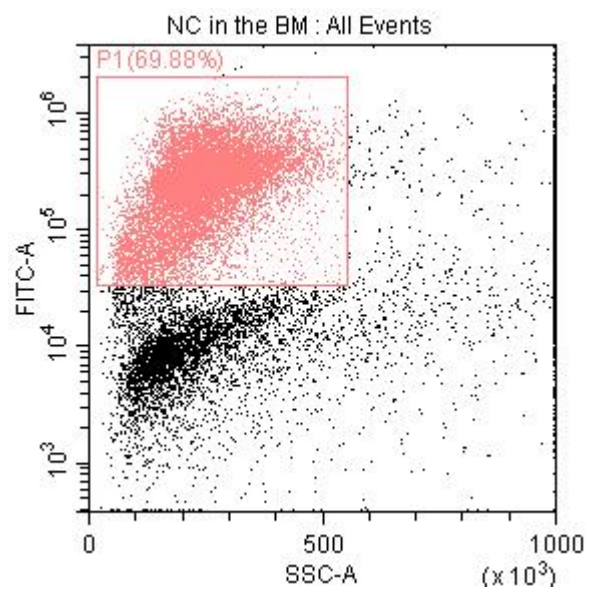

**E**

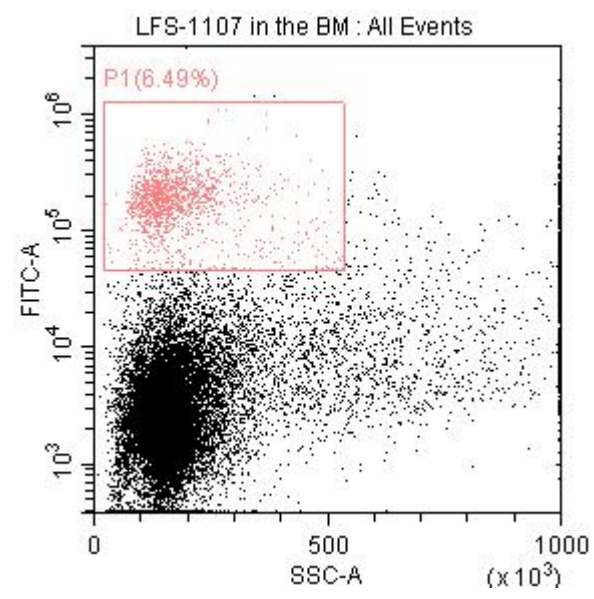

Supplement: Figure 3—source data 3. [file elife-80625-fig3-data3.zip › Figure 3-source data 3/Figure 3-source data C-E.pdf]

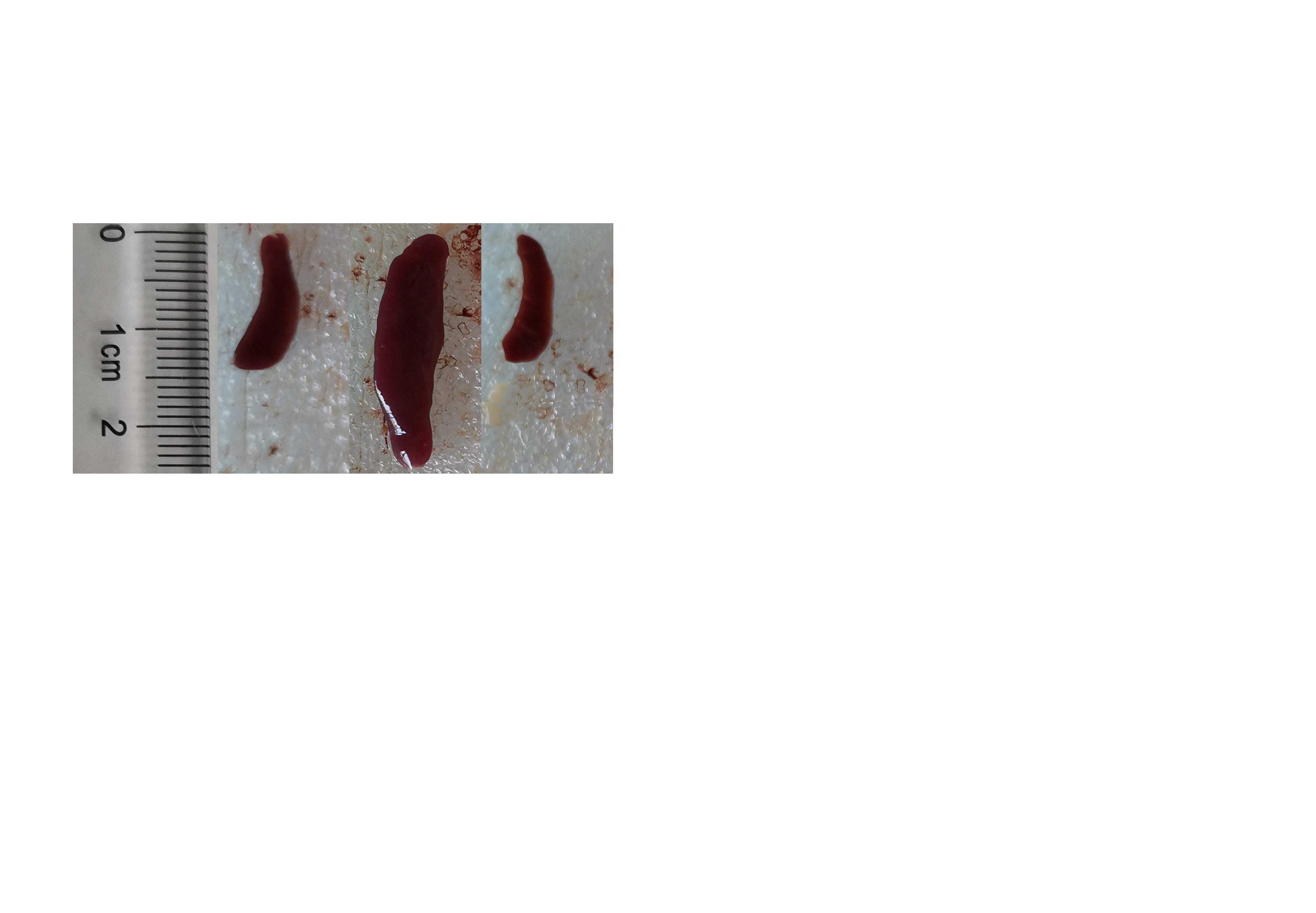

Supplement: Figure 3—source data 4. [file elife-80625-fig3-data4.zip › Figure 3-source data 4/Figure 3-source data F.jpg]

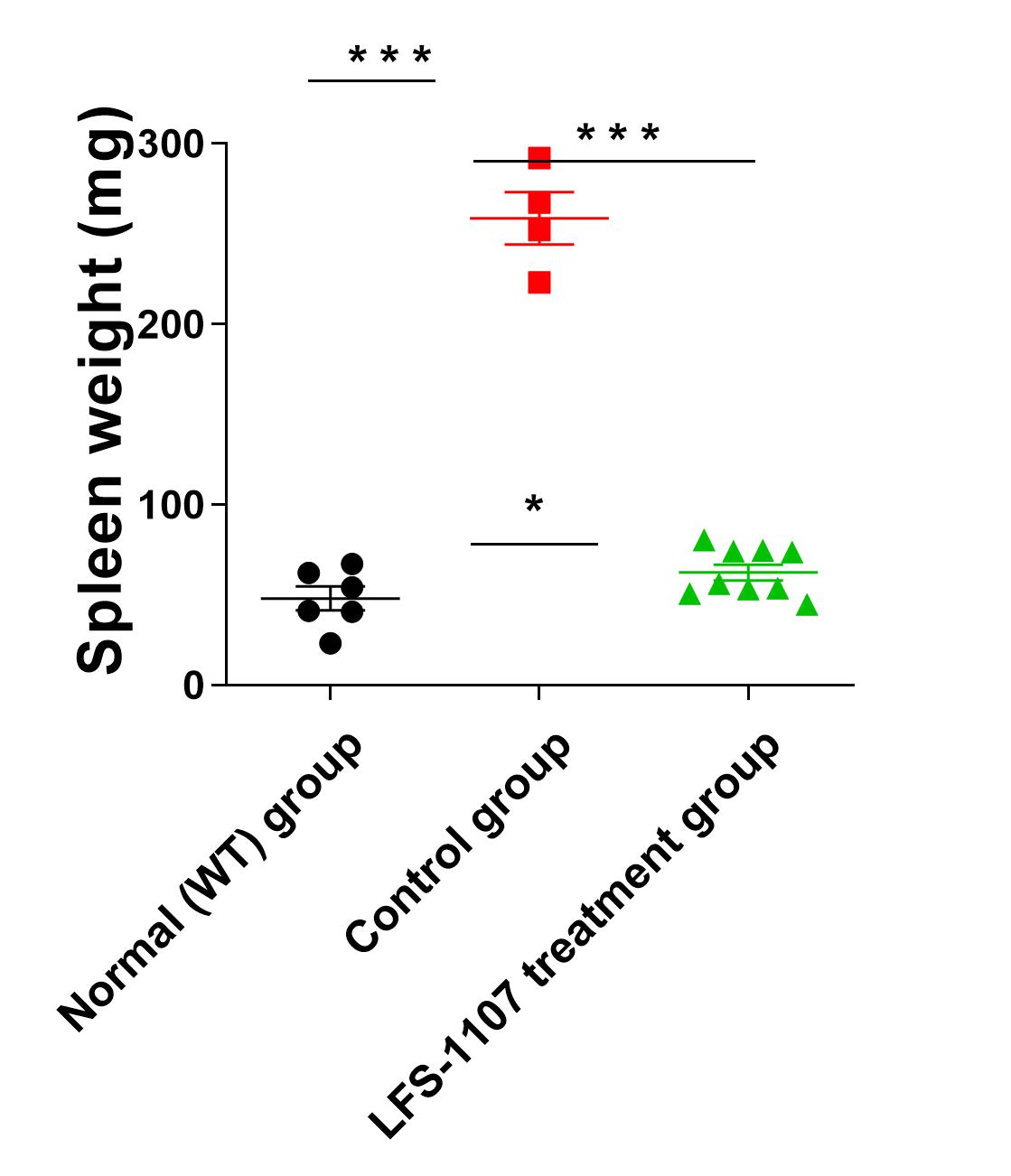

Supplement: Figure 3—source data 5. [file elife-80625-fig3-data5.zip › Figure 3-source data 5/3G.jpg]

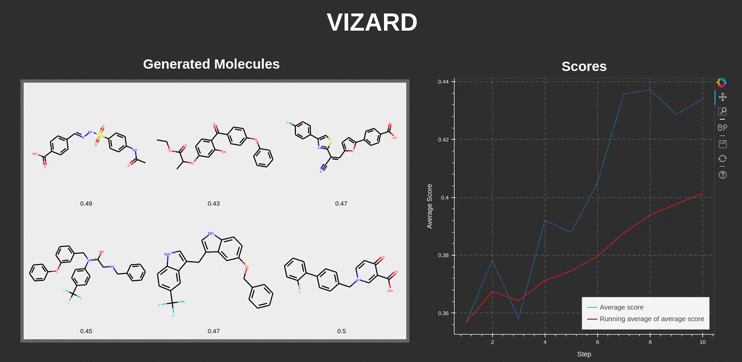

Supplement: Source code 1. [file elife-80625-code1.zip › Source_codes/images/celecoxib_analogues.gif]
